# Fetal Metabolomic Alterations Following Porcine Reproductive and Respiratory Syndrome Virus Infection

**DOI:** 10.3389/fmolb.2020.559688

**Published:** 2020-12-11

**Authors:** Carolina M. Malgarin, Daniel J. MacPhee, John C. S. Harding

**Affiliations:** ^1^Department of Large Animal Clinical Sciences, Western College of Veterinary Medicine, Saskatoon, SK, Canada; ^2^Department of Veterinary Biomedical Sciences, Western College of Veterinary Medicine, Saskatoon, SK, Canada

**Keywords:** PRRS, reproductive, pig, metabolome, kynurenine, fetal death, intrauterine growth retardation (IUGR), disease progression

## Abstract

PRRSV infection in third-trimester pregnant sows can lead to fetal death and abortions, although the mechanisms triggering these effects are not well understood. Since resistant and susceptible fetuses can coexist in the same litter, we propose that there may be differential mechanisms used by some fetuses to evade infection and/or disease progression. Our objectives were to investigate possible differences in the metabolome of PRRSV-infected and non-infected fetuses, as well as the interaction of altered intrauterine growth development and PRRSV infection to elucidate possible causes of fetal death following PRRSV infection. Near-term serum samples collected from fetuses on gestation day 106, 21 days post PRRSV-2 infection, were processed by direct flow injection mass spectrometry (DI-MS) and nuclear magnetic resonance (NMR) techniques. Experiment one investigated disease progression with 24 fetuses selected from each of four phenotypic groups: fetuses from non-inoculated gilts (CTRL); fetuses from inoculated gilts that escaped infection (UNINF); infected high viral load viable fetuses (INF); and infected high viral load meconium-stained fetuses (MEC). Experiment two investigated the interaction of intrauterine growth retardation (IUGR) and PRRSV infection by analyzing differences among: non-infected normal development (CON-N); CON-IUGR; PRRS infected normal development (PRRS-N); and PRRS-IUGR. Univariate and multivariate (PCA, PLS-DA) statistics determined group differences among various contrasts, and the most important metabolites associated with disease progression and fetal development. Significant differences in the metabolome were observed, especially between PRRSV-negative fetuses (CTRL and UNINF) and MEC fetuses, while INF fetuses appear to span both groups. The two metabolites with highest variable importance in projection (VIP) scores related to disease progression were alpha-aminoadipic acid (alpha-AAA) and kynurenine (KYN), having the highest concentration in MEC and INF fetuses, respectively, compared to CTRL and UNINF. In experiment two, non-IUGR fetuses were found to have increased levels of lysoPCs, PCs and amino acids compared to IUGR fetuses, while the near complete absence of lysoPCs and PCs in IUGR fetuses, even during infection, indicate a distinctive response to infection compared to non-growth retarded fetuses. Possible markers of PRRSV fetal susceptibility, such as alpha-AAA, kynurenine and lysoPCs, are presented and discussed.

## Introduction

In medical research, the development of global untargeted metabolomic techniques has provided tremendous insight into the cellular and biochemical processes occurring in healthy individuals, or those subjected to physiological stresses, neoplasia or infective diseases. The unique value stems from the fact that, unlike other “omic” approaches like genomics, transcriptomics or proteomics, metabolomics provides a tool to directly measure biochemical response (Patti et al., 2012). While metabolomics has been used in swine-related nutritional and reproductive research, the technique is less commonly employed to study host responses to veterinary infective diseases, and porcine diseases in particular. Although the utility of this approach lies primarily in hypothesis generation, recent studies have shed light on possible metabolic and physiologic changes following respiratory disease caused by *Mycoplasma hyopneumoniae* (Nair et al., 2019), enteric disease cause by *Brachyspira hyodysenteriae* (Welle et al., 2017) and a potential interaction between the host and gut microbiome in the development of disease following infection with classical swine fever virus (Gong et al., 2017).

Porcine reproductive and respiratory syndrome (PRRS) is a financially devastating Arteriviral disease (Holtkamp et al., 2013) associated with systemic vasculitis, immunosuppression, and persistent infections in post-natal pigs. Infection of pregnant sows or gilts during the third trimester results in rapid endometritis and endometrial vasculitis (Novakovic et al., 2018) followed by rapid transmission across the diffuse epitheliochorial placenta within days of maternal infection (Suleman et al., 2018; Malgarin et al., 2019). Fetal infection can lead to death and abortions, however, there is vast heterogeneity in outcome within and between litters (Ladinig et al., 2014d). Pregnant gilts experience minor symptoms but have a significant endometritis, vasculitis and immune response following PRRSV infection (Ladinig et al., 2014b,c; Novakovic et al., 2016) that may impact the efficiency of the placenta and thereby alter fetal metabolomics and compromise growth and viability. The mechanisms that trigger fetal death, as well as factors associated with variation in fetal susceptibility or resilience to viral infection, are not well understood. Since resilient and susceptible fetuses can coexist in the same litter, differential physiological mechanisms might underlie the ability of some fetuses to evade infection or resist disease progression. For example, previous large-scale research allowing the categorization of fetuses according to their phenotypic responses identified differences in genomic (e.g., fetal SNPs related to thymus viral load and fetal viability) (Yang et al., 2016) and transcriptomic (e.g., increased TREM1 signaling as disease progresses) (Wilkinson et al., 2016) profiles of susceptible and resilient fetuses, indicating that events occurring within the fetal compartment are important to the final outcome. Moreover, it has been observed that intrauterine growth-retarded (IUGR) fetuses, fetuses born with lower body weight relative to brain weight (i.e., “brain sparing”) when compared to its siblings, possess lower viral concentration after maternal inoculation than fetuses experiencing normal intrauterine growth (Ladinig et al., 2014a).

In spite of being a difficult compartment to access, understanding changes in the fetal metabolome may help explain the pathophysiology associated with disease progression and fetal death following both maternal and fetal PRRSV infection. Metabolomics may also help to identify differences between susceptible and resistant fetuses, including why IUGR fetuses appear to be more resistant to infection. With this background, the aim of this study was to identify key differences in the metabolomic profiles of fetuses representative of unique phenotypic susceptibility groups following maternal PRRSV infection, and observe how retarded fetal development may alter the metabolic profiles of PRRSV-infected fetuses.

## Materials and Methods

The project was approved by the University of Saskatchewan's Animal Research Ethics Board and adhered to the Canadian Council on Animal Care guidelines for humane animal use (permit #20110102).

The experimental protocol has been extensively described (Ladinig et al., 2014d). Briefly, 114 pregnant gilts were inoculated with PRRSV2 (NVSL 97-7895) at day 85 of gestation while 19 pregnant gilts were mock inoculated negative controls. Animals were humanely euthanized 21 days post inoculation (DPI), the gravid reproductive tract removed, and fetuses categorized as per their preservation status as: viable (VIA), meconium-stained (MEC), decomposed (DEC), and autolyzed (AUT). Blood was collected via the axillary artery from VIA and MEC fetuses and refrigerated after clot formation. Serum was separated the following morning then frozen at −80°C pending further analysis. The PRRSV RNA concentration in fetal sera and thymus was quantified using an in-house RT-qPCR targeting NVSL 97-7895 and reported as logarithm base 10 DNA copies per μL sera or mg tissue.

### Phenotypic Fetal Susceptibility Groups

#### Experiment 1 - Disease Progression

A subset of sera from fetuses representing four phenotypic severity groups (*n* = 24/group) were selected for untargeted metabolomic analyses as follow: (1) fetuses from non-inoculated control gilts (CTRL); (2) fetuses that escaped infection from PRRSV inoculated gilts, i.e., negative viral load (UNINF); (3) viable PRRSV-infected fetuses with high viral load (over 3.5 log_10_; mean 6.7 ± 0.9 genome copies/mg) in fetal thymus (HVL-VIA); and (4) PRRSV-infected meconium-stained fetuses with high viral load (over 5 log_10_; mean 7.3 ± 0.9 genome copies/mg) (MEC). A first batch of samples (48 samples; 12 per group) was submitted to The Metabolomics Innovation Centre (TMIC) at the University of Alberta to initially determine the within group variability across metabolites. This data was used to establish the sample size required and a second batch (48 samples; 12 per group) was submitted and tested subsequently. Fetuses from PRRSV-inoculated groups were randomly selected blocking by litter (one UNINF, HVL-VIA and MEC fetus from each of 24 litters). The CTRL group comprised of one randomly selected fetus from each of 19 non-infected gilts plus a second fetus from each of 5 randomly selected non-inoculated litters (24 in total). All serum samples submitted to the TMIC had been previously used to measure PRRSV RNA concentration, thus, had been thawed/re-frozen up to 3 times, admittedly a weakness of this study.

#### Experiment 2 – Fetal IUGR

A subset of fetuses investigating the interaction of intrauterine growth and maternal PRRSV infection status was selected using a 2 x 2 factorial design: (1) Non-IUGR (normal development) fetuses from non-inoculated control gilts (CON-N, *n* = 23); (2) IUGR fetuses from non-inoculated control gilts (CON-IUGR, *n* = 24); (3) Non-IUGR fetuses from PRRSV-infected gilts (PRRS-N, *n* = 25); and (4) IUGR fetuses from PRRSV-infected gilts (PRRS-IUGR, *n* = 24). IUGR was characterized based on the fetal brain:liver weight ratio as previously described (Ladinig et al., 2014a), with high brain:liver ratio representative of the brain sparing effect typifying IUGR fetuses. The IUGR and non-IUGR fetuses were selected at the opposing extreme ends of the brain:liver range, and averaged 1.75 ± 0.52 and 0.64 ± 0.11, respectively. The PRRSV infected fetuses had mean viral load of 4.7 ± 3.2 log_10_ RNA copies, however, not all fetuses in the PRRSV-IUGR group were infected (discussed below in more detail). Similar to experiment 1, an initial batch of sera (48 samples; 12 per group) was submitted to TMIC for the purpose of estimating the sample size, then a second batch of equal size was subsequently submitted. Due to the relative sparsity of IUGR fetuses, they were selected from 28 PRRSV-infected and 19-non-infected litters (1–4 fetuses/litter) while including as many IUGR/non-IUGR matched littermates as possible. The final section included 33/47 matched control and 30/49 matched inoculated fetuses. All samples had been thawed/re-frozen up to three times for reasons describe above.

### Metabolomic Techniques

Nuclear Magnetic Resonance (NMR) and Direct Flow Injection Mass Spectrometry (DI-MS) were performed following each submission to TMIC. The results from identical metabolites found in both assays were averaged.

#### Sample Preparation, NMR Spectroscopy and Compound Quantification and Identification

To remove large molecular weight proteins and lipoproteins from the sera, which affects the identification of the small molecular weight metabolites by NMR spectroscopy, a deproteinization step involving ultra-filtration (Psychogios et al., 2011) was undertaken. Prior to filtration, the 3 KDa cut-off centrifugal filter units (Amicon Microcon YM-3) were rinsed five times each with 0.5 mL ultrapure water and centrifuged (13,000 × g for 10 min) to remove residual glycerol bound to the filter membranes. Aliquots of each serum sample were then transferred into the centrifuge filter devices and spun (13,000 × g for 20 min) to remove macromolecules from the sample. The filtrates were checked visually for any evidence of a compromised membrane and if observed the filtration process was repeated. The filtrates were collected and if the total volume was <600 μL an appropriate amount of 50 mM NaH_2_PO_4_ buffer (pH 7) was added to bring the total volume to 600 μL. For such samples, the dilution factor was used to correct the metabolite concentrations in the subsequent analysis. Subsequently, 70 μL of deuterium oxide (D2O) and 30 μL of buffer solution (12 mM disodium−2, 2-dimethyl-2-silcepentane-5- sulphonate, 730 mM imidazole, and 0.47% NaN_3_ in water) were added to the samples. The sample (700 μL) was transferred to a standard NMR tube for subsequent spectral analysis. All 1H-NMR spectra were collected on a 500 MHz Inova (Varian Inc. Palo Alto, CA) spectrometer equipped with a 5 mm HCN Z-gradient pulsed-field gradient (PFG) room-temperature probe. 1H-NMR spectra were acquired at 25°C using the first transient of the NOESY- pre-saturation pulse sequence, chosen for its high degree of quantitative accuracy (Saude et al., 2006). All free induction decays (FID's) were zero-filled to 64 K data points and subjected to line broadening of 0.5 Hz. The singlet produced by the DSS methyl groups was used as an internal standard for chemical shift referencing (set to 0 ppm) and for quantification. All 1H-NMR spectra were processed and analyzed using the Chenomx NMR Suite Professional Software package version 7.1 (Chenomx Inc., Edmonton, AB). The Chenomx NMR Suite software allows for qualitative and quantitative analysis of an NMR spectrum by manually fitting spectral signatures from an internal database to the spectrum. Specifically, the spectral fitting for metabolite was done using the standard Chenomx 500 MHz metabolite library. Typically, 90% of visible peaks were assigned to a compound and more than 90% of the spectral area could be routinely fit using the Chenomx spectral analysis software. Most visible peaks were annotated with a compound name previously shown to provide absolute concentration accuracy of >90%. Each spectrum was processed and analyzed by at least two NMR spectroscopists to minimize compound misidentification and miss-quantification.

#### Combined Direct Flow Injection and LC-MS/MS Compound Identification and Quantification

An untargeted quantitative approach using a combination of direct injection mass spectrometry (AbsoluteIDQ® Kit) with a reverse-phase LC-MS/MS Kit (BIOCRATES Life Sciences AG, Austria) was used. This kit was selected based on its capability to identify and quantify up to 180 different endogenous metabolites including amino acids, acylcarnitines, biogenic amines, glycerophospholipids, sphingolipids and sugars. Serum samples were analyzed with the AbsoluteIDQ kit as per the manufacturer's instructions. Briefly, samples were vortexed then centrifuged at 13,000 × g for 10 min. Ten microliter was loaded onto the center of the filter on the upper 96-well kit plate and dried in a stream of nitrogen. Subsequently, 20 μL of a 5% solution of phenyl-isothiocyanate was added for derivatization. After incubation, the filter spots were dried using an evaporator. Extraction of metabolites was achieved by adding 300 μL methanol containing 5 mM ammonium acetate. Extracts were obtained by centrifugation into the lower 96-deep well plate, followed by dilution with kit MS running solvent. Mass spectrometric analysis was performed on an API4000 Qtrap® tandem mass spectrometry instrument (Applied Biosystems/MDS Analytical Technologies, Foster City, CA) equipped with a solvent delivery system. The samples were delivered to the mass spectrometer by a LC method followed by a direct injection (DI) method. The Biocrates MetIQ software was used to control the assay workflow, including sample registration, calculation of metabolite concentrations and export of data for analysis. A targeted profiling scheme was used to quantitatively screen for known small molecule metabolites using multiple reaction monitoring, neutral loss and precursor ion scans. Forty different isotope-labeled internal standards (representing amino acids and biogenic amines) at 7 concentrations were used to create the calibration curves. Regression was used to calculate the *R*^2^ for six repetitive preparations, which resulted in *R*^2^ equal or higher than 0.990 for most metabolites. Accuracy of the measurements ranged between 80 and 115% for all analyses, while intra-day precision and accuracy of the standards remained <16% CV and ranging from 57 to 120%, respectively. Reproducibility of the assay was examined and scored between 4 and 37% CV, while inter-day accuracy ranged between 51 and 159%. All metabolites discussed herein are considered level 1 Identified Metabolites.

### Statistical and Pathway Analyses

Differences in metabolite levels resulting from running the two separate batches were accounted for by quality control (QC) samples and then centralizing the data (i.e., differences in the means of batches 1 and 2 for each metabolite were added to the batch 2 subject values). Thereafter, the analytical platform MetaboAnalyst (https://www.metaboanalyst.ca/) was used for all the statistical analyses. Data normalization was completed by log base 10 transformation and Pareto scaling followed by visualization of the normalized data using box plots and kernel density plots. Shapiro-Wilk tests were performed and because a proportion of the compounds were not normally distributed non- parametric univariate analysis was used to screen for the most significant (FDR < 0.01) metabolites to be included in multivariate analyses to identify metabolite alterations associated with each of the four phenotypic groups in each experiment.

MetaboAnalyst was also used to generate the pathway analysis plots, which combine the topography (Y axis) and pathway impact (X axis) analyses. The plots were re-formatted in R to also display the relative number of compounds in each pathway (bubble size) and the percentage of hits we detected in that pathway (bubble color scale). Pathways with *P*-values (log base10) and “pathway impact” higher than the mid-point for that contrast were labeled. The data generated from MetaboAnalyst was also used to produce boxplots of the significant (*P*-value < 0.05) compounds leading those pathways.

Group differences were visualized using scatter plots following: (a) unsupervised Principal Components Analysis (PCA) that maximized the data variance into few principal components; and (b) supervised Partial Least Squares Discriminant Analysis (PLS-DA) that generated principle components maximizing variation among the phenotypic groups. PLS-DA model quality was assessed by 10-fold cross-validation, generation of the *R*^2^ and *Q*^2^ measures, and assessing the significance by permutations tests (2,000 permutations). Variable Importance in Projection (VIP) scores were calculated to provide a relative ranking of metabolites, and the 15 most important metabolites responsible for group differences were further examined. A targeted pathway analysis was performed on the metabolites or groups of metabolites with VIP scores >1. The Human Metabolome Database (http://www.hmdb.ca/) and the Kyoto Encyclopedia of Genes and Genomes (https://www.genome.jp/kegg/) were used to identify biochemical pathways specific to the high VIP metabolites. Those were followed by an extensive literature review to relate the most significant metabolites to their biological reactions, focusing especially on possible PRRS, IUGR and infectious diseases related pathways.

## Results

### Metabolomic Profiles Associated With Disease Progression

**Figure 1:**
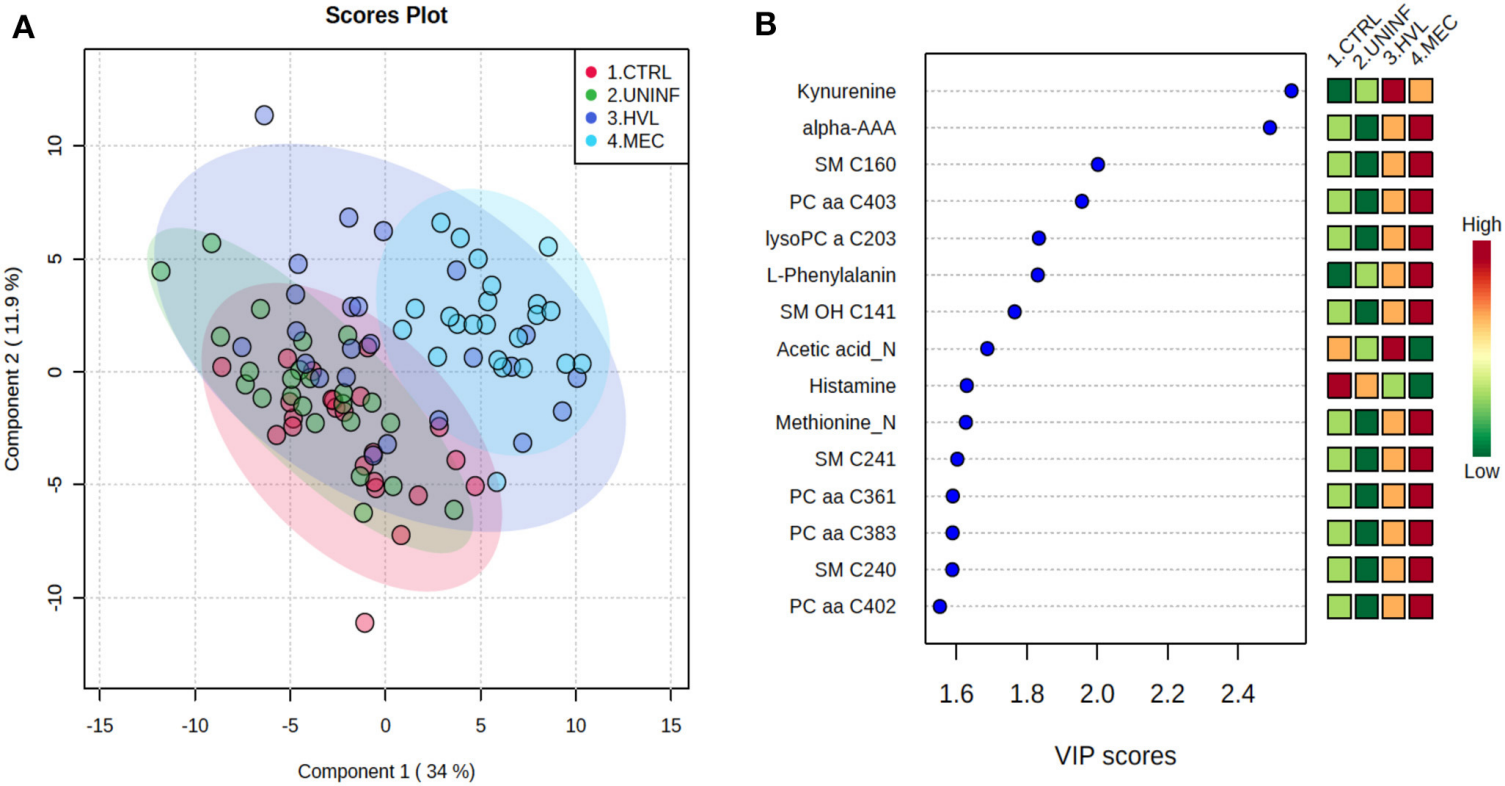
Metabolite profiles of four disease progression fetal phenotypes. **(A)** Two component PLS-DA score plot of all groups with individual fetuses represented by dots. **(B)** Variance Importance in Projection (VIP) score plot displaying the 15 most important metabolites differentiating the groups with colored side bar displaying the relative metabolite concentration in each group. 1.CTRL: fetuses from non-inoculated control gilts; 2.UNINF: fetuses from PRRSV inoculated gilts that escaped infection, i.e., negative viral load; 3.HVL-VIA: viable PRRSV-infected fetuses with high viral load (over 3.5 log_10_; mean 6.7 ± 0.9 genome copies/mg) in fetal thymus; and 4.MEC: PRRSV-infected meconium-stained fetuses with high viral load (over 5 log_10_; mean 7.3 ± 0.9 genome copies/mg). Alpha-AAA, alpha-aminoadipic acid; lysoPC, lysophosphocholine; PC, phosphocholine; SM, sphingomyelin.

In total, 140 common metabolites (DI-MS: 108, NMR: 32) were detected. Seventy-eight were phosphocholines (PC) or lysophosphocholines (lysoPC), 22 were amino acids (AA), 15 were sphingomyelins (SM), and 25 were in other categories. Of these, 89 were identified by Kruskal-Wallis as having significant differences (FDR < 0.01) among groups. Across all metabolites, profiles of the UNINF and MEC groups were the most distinct while being unique from each other, with metabolites largely positive/increased for MEC and negative/decreased for UNINF. The same pattern was observed following analyses by PCA and PLS-DA (2 components, *R*^2^ = 0.68, *Q*^2^ = 0.54) where clear differences (*P* < 5e-04; 0/2,000 permutations) in the metabolome were observed for the PRRSV negative groups (CTRL and UNINF) compared to the MEC fetuses, while the metabolome of HVL-VIA fetuses spanned both groups (Figure 1A). The metabolites with the greatest contribution (highest VIP scores) to disease progression and group separation were kynurenine and aminoadipic acid (alpha-AAA), being of highest concentration in MEC and HVL-VIA fetuses compared to UNINF and CTRL (Figure 1B). Thirteen other metabolites including amino acids (2), sphingomyelins (SMs; 4), phosphocholines (PCs; 4), lysophosphocholines (lysoPCs;1), acetic acid and histamine had VIP scores > 1.5 and were significantly different among groups. Levels were lower in PRRSV-negative fetuses compared to infected fetuses (HVL-VIA, MEC) with the exception of histamine and acetic acid. Individual group contrasts are described in more detail below and Table 1 contains all the important metabolites indicating their significance, VIP score, and fold change (FC) between groups for each of the contrasts.

**Table 1:** Metabolites with significant differences among fetal PRRS disease progression groups.

**Contrasts**	**CTRL vs. UNINF**			**UNINF vs. HVL-VIA**			**HVL-VIA vs. MEC**		
**Metabolites**	**VIP score**	**FDR**	**FC**	**VIP score**	**FDR**	**FC**	**VIP score**	**FDR**	**FC**
ADMA	<1	ns	ns	1.58	0.025	ns	<1	ns	ns
Alpha-AAA	3.39	0.019	2.675	3.72	0.001	0.17	1.55	0.018	ns
C2	<1	ns	ns	1.72	0.012	ns	<1	ns	ns
Creatine	<1	ns	ns	1.34	0.012	ns	<1	ns	ns
Formate	1.09	ns	ns	2.08	<0.001	ns	<1	ns	ns
Histamine	<1	ns	ns	<1	ns	ns	1.38	0.003	ns
Kynurenine	<1	ns	ns	3.19	<0.001	0.19	<1	ns	ns
L-Glutamic acid	1.40	0.019	ns	<1	ns	ns	<1	0.006	ns
L-Phenylalanine	<1	ns	ns	1.69	0.001	ns	<1	ns	ns
L-Threonine	1.48	0.027	ns	<1	ns	ns	<1	ns	ns
lysoPC a C160	1.47	ns	ns	<1	ns	ns	1.00	0.017	ns
lysoPC a C170	1.63	0.035	ns	<1	ns	ns	<1	0.025	ns
lysoPC a C180	1.37	ns	ns	<1	ns	ns	1.28	0.004	ns
lysoPC a C181	1.22	ns	ns	<1	ns	ns	1.43	0.003	ns
lysoPC a C203	<1	ns	ns	1.16	ns	ns	1.44	0.003	ns
Methionine	1.06	0.043	ns	1.98	<0.001	ns	<1	ns	ns
PC aa C360	1.35	0.030	ns	<1	ns	ns	<1	0.007	ns
PC aa C361	<1	ns	ns	<1	ns	ns	1.31	0.003	ns
PC aa C363	<1	ns	ns	<1	ns	ns	1.27	0.006	ns
PC aa C366	1.54	0.027	ns	<1	ns	ns	<1	ns	ns
PC aa C383	<1	ns	ns	<1	ns	ns	1.32	0.003	ns
PC aa C386	1.41	0.043	ns	<1	ns	ns	1.25	0.006	ns
PC aa C403	<1	ns	ns	1.08	ns	ns	1.46	0.003	ns
PC aa C404	<1	ns	ns	<1	ns	ns	1.40	0.005	ns
PC aa C405	<1	ns	ns	<1	ns	ns	1.34	0.005	ns
PC aa C406	1.30	ns	ns	<1	ns	ns	1.26	0.006	ns
PC ae C380	1.43	0.035	ns	<1	ns	ns	1.25	0.009	ns
PC ae C386	1.31	0.043	ns	<1	ns	ns	<1	0.032	ns
PC ae C401	1.70	0.019	ns	<1	ns	ns	1.13	0.014	ns
PC ae C406	1.41	0.035	ns	<1	ns	ns	<1	0.007	ns
PC ae C422	1.30	ns	ns	<1	ns	ns	1.26	0.007	ns
PC ae C423	1.49	0.035	ns	<1	ns	ns	1.15	0.007	ns
SM C160	<1	ns	ns	1.62	0.025	ns	1.25	0.004	ns
SM C161	<1	ns	ns	1.23	ns	ns	1.12	0.005	ns
SM C180	<1	ns	ns	1.29	0.039	ns	1.02	0.005	ns
SM C240	<1	ns	ns	1.26	0.047	ns	1.12	0.004	ns
SM C241	<1	ns	ns	1.30	0.049	ns	1.05	0.005	ns
SM OH C141	<1	ns	ns	1.43	ns	ns	1.17	0.006	ns
SM OH C222	1.01	ns	ns	1.22	ns	ns	<1	0.030	ns

*FC, fold change; FDR, false discovery rate; VIP, Variable Importance in Projection; ns, not significant*.

#### Control vs. Uninfected Fetuses

**Figure 2:**
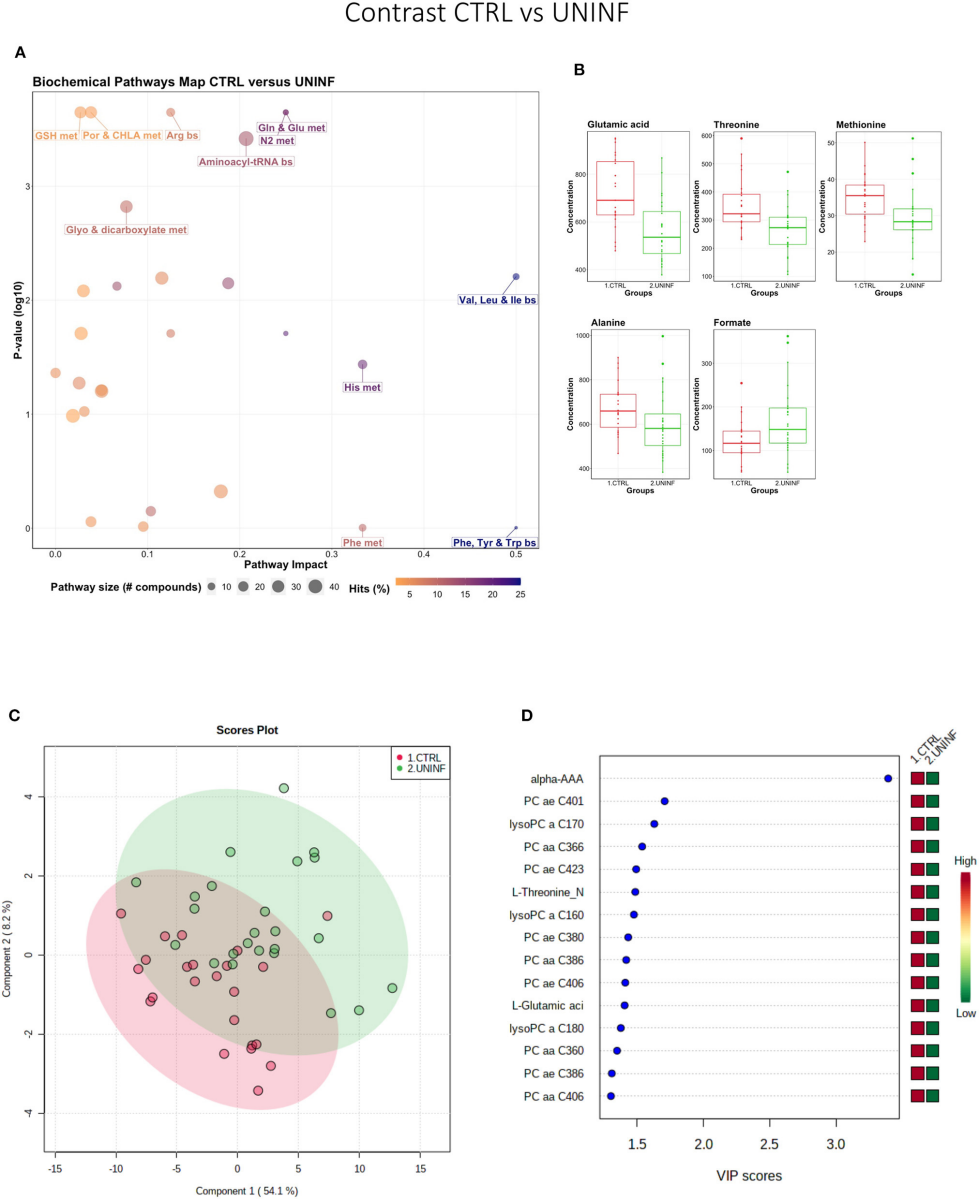
Metabolite profile of control (CTRL) and uninfected (UNINF) contrast. **(A)** Biochemical pathway plot displaying the most significant (Y-axis; *P*-value < 0.05) and impactful (X-axis) pathways distinguishing the groups. Size of dot represents the size of the pathway (number of compounds). Dark colors (from light orange to dark purple) represent higher hits (percentage of the pathway detected in our study). **(B)** Boxplots of the concentration of significant (*P*-value < 0.05) compounds distinguishing the groups. **(C)** Two component PLS-DA score plots with individual fetuses represented by dots. **(D)** Variance Importance in Projection (VIP) score plots displaying the 15 most important metabolites differentiating groups with colored side bar displaying the relative metabolite concentration in each group. 1.CTRL: fetuses from non-inoculated control gilts; 2.UNINF: fetuses from PRRSV inoculated gilts that escaped infection, i.e., negative viral load. Met, metabolism; bs, biosynthesis; deg, degradation; Por, Porphyrin; CHLA, chlorophyll; Glyo, Glyoxylate; Buta, Butanoate; GSH, Glutathione; GPL, Glycerophospholipid; ALA, alpha-Linoleic acid; ARA, arachidonic acid; SeC, Selenocompound; Prop, Propanoate; Alpha-AAA, alpha-aminoadipic acid; lysoPC, lysophosphocholine; PC, phosphocholine.

This contrast aimed to investigate the impact of maternal infection only and response by non-infected fetuses on fetal parameters. Pathways related to amino acid metabolism or biosynthesis were most impactful and/or significant (Figure 2A) with a number of key amino acids decreased in the UNINF group (Figure 2B). The global metabolomes of CTRL and UNINF fetuses trended toward significance (*P* = 0.075; 145/2,000 permutations) based on PLS-DA (3 components, *R*^2^ = 0.62, *Q*^2^ = 0.27) showing marginal differences between groups (Figure 2C). As this contrast reflects fetal responses following PRRSV infection of the maternal-fetal interface (MFI), the differences although minimal, indicate that some fetuses are responding to endometrial infection without themselves being infected. The most important in variance metabolites between the CTRL and UNINF groups based on VIP score (although most are in between 1 and 2, not consider highly important) were alpha-AAA, amino acids (glutamic acid and threonine), lysoPCs (3), and some PCs (9). Alpha-AAA stood out as the most contributory metabolite distinguishing these groups (Figure 2D) and the only metabolite with a VIP score over 2.0. It was at higher concentration in the CTRL group.

#### Uninfected vs. High Viral Load Viable Fetuses

**Figure 3:**
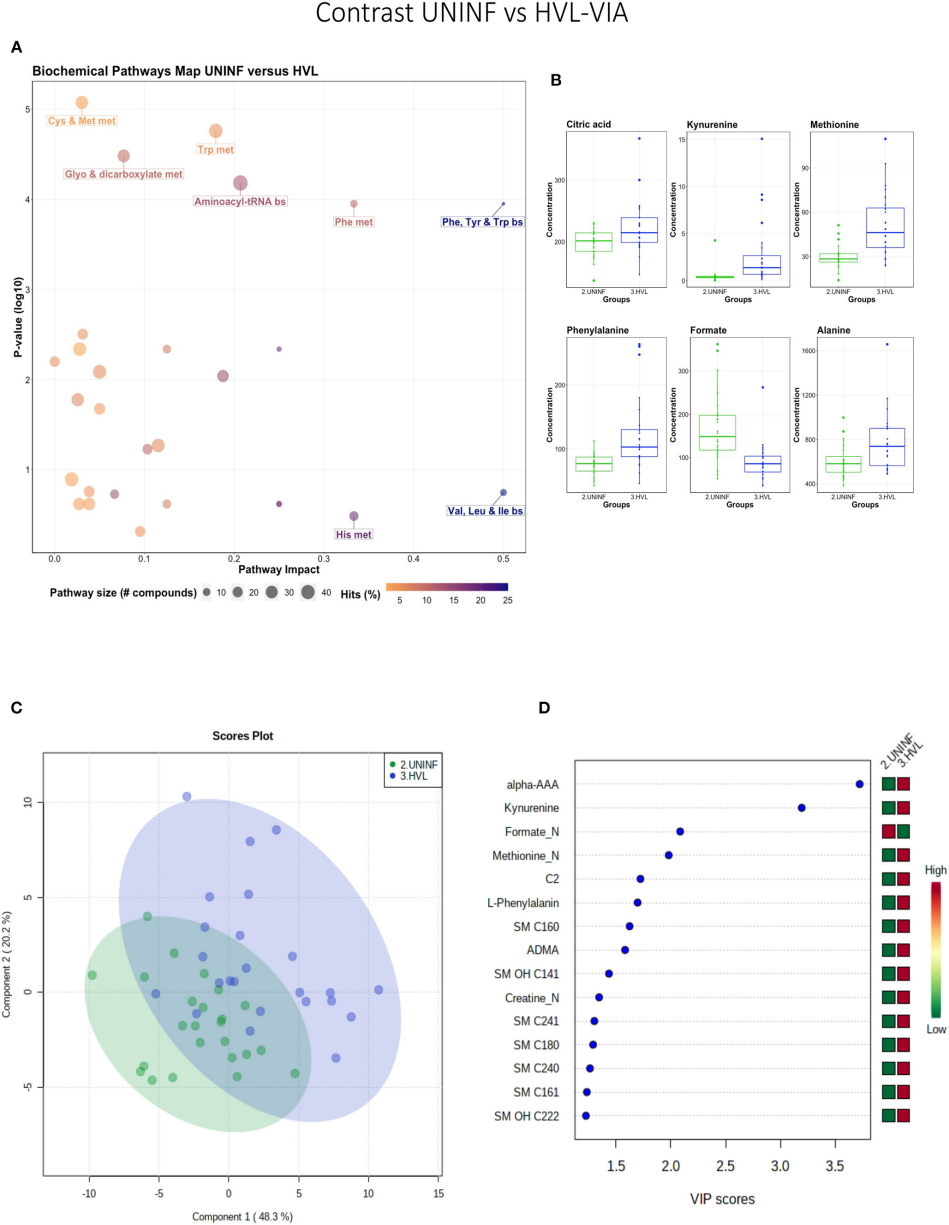
Metabolite profile of uninfected (UNINF) and high-viral load (HVL-VIA) contrast. **(A)** Biochemical pathway plot displaying the most significant (Y-axis; *P*-value < 0.05) and impactful (X-axis) pathways distinguishing the groups. Size of dot represents the size of the pathway (number of compounds). Dark colors (from light orange to dark purple) represent higher hits (percentage of the pathway detected in our study). **(B)** Boxplots of the concentration of significant (*P*-value < 0.05) compounds distinguishing the groups. **(C)** Two component PLS-DA score plots with individual fetuses represented by dots. **(D)** Variance Importance in Projection (VIP) score plots displaying the 15 most important metabolites differentiating groups with colored side bar displaying the relative metabolite concentration in each group. 2.UNINF: fetuses from PRRSV inoculated gilts that escaped infection, i.e., negative viral load; 3.HVL-VIA: viable PRRSV-infected fetuses with high viral load (over 3.5 log_10_; mean 6.7 ± 0.9 genome copies/mg) in fetal thymus. Met, metabolism; bs, biosynthesis; deg, degradation; Por, Porphyrin; CHLA, chlorophyll; GSH, Glutathione; Glyo, Glyoxylate; Buta, Butanoate; ALA, alpha-Linoleic acid; ARA, arachidonic acid; SeC, Selenocompound; Prop, Propanoate; ADMA, asymmetric dimethylarginine; Alpha-AAA, alpha-aminoadipic acid; C2, acetylcarnitine; SM, sphingomyelin.

This contrast was made to highlight the fetal response to PRRSV infection. Pathways mainly related to amino acid metabolism, but also sphingolipid metabolism, were most prominent in this contrast (Figure 3A), led by the compounds shown in Figure 3B including kynurenine. Group variation was much greater and significantly different (*P* < 5e-04; 0/2,000 permutations) in the HVL-VIA compared to the UNINF group (Figure 3C) on PLS-DA (2 components, *R*^2^ = 0.52, *Q*^2^ = 0.41), likely reflecting the heterogeneity within the HVL-VIA group in terms of individual variability in response or timing of fetal infection, or both. Infection of the fetus was mostly related to increased levels of kynurenine, alpha-AAA, acylcarnitine (C2), formate, amino acids (methionine, phenylalanine, and creatine), and asymmetric dimethylarginine (ADMA) among others. In addition to alpha-AAA, kynurenine also stood out as an important metabolite (VIP score >3) distinguishing the HVL-VIA group (Figure 3D). With the exception of formate, all of the most important metabolites distinguishing these two groups were at greater concentration in the HVL-VIA group.

#### High Viral Load Viable vs. Meconium-Stained Fetuses

**Figure 4:**
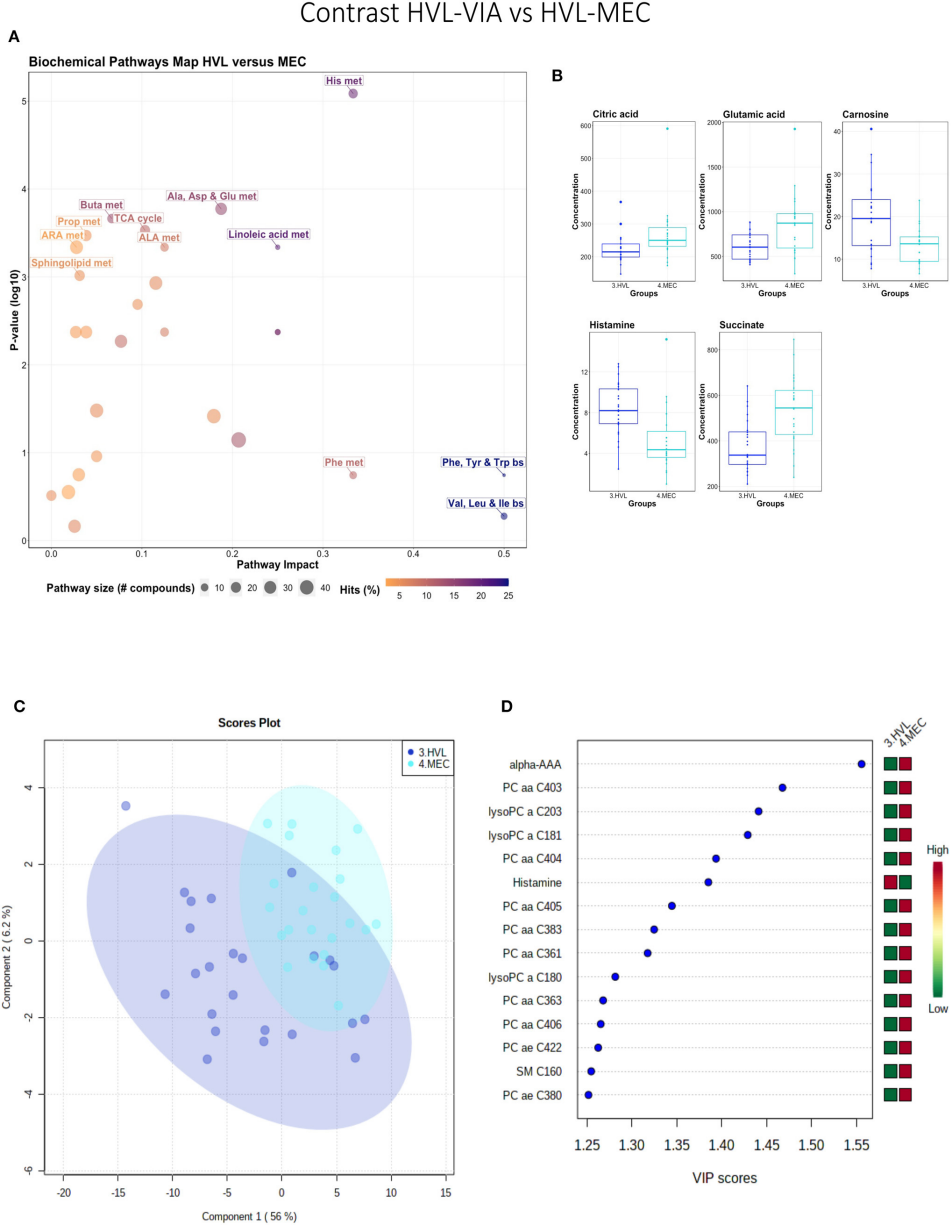
Metabolite profile of high-viral load (HVL-VIA) and meconium-stained fetuses (MEC) contrast. **(A)** Biochemical pathway plot displaying the most significant (Y-axis; *P*-value < 0.05) and impactful (X-axis) pathways distinguishing the groups. Size of dot represents the size of the pathway (number of compounds). Dark colors (from light orange to dark purple) represent higher hits (percentage of the pathway detected in our study). **(B)** Boxplots of the concentration of significant (*P*-value < 0.05) compounds distinguishing the groups. **(C)** Two component PLS-DA score plots with individual fetuses represented by dots. **(D)** Variance Importance in Projection (VIP) score plots displaying the 15 most important metabolites differentiating groups with colored side bar displaying the relative metabolite concentration in each group. 3.HVL-VIA: viable PRRSV-infected fetuses with high viral load (over 3.5 log_10_; mean 6.7 ± 0.9 genome copies/mg) in fetal thymus; 4.MEC: PRRSV-infected meconium-stained fetuses with high viral load (over 5 log_10_; mean 7.3 ±0.9 genome copies/mg). Met, metabolism; bs, biosynthesis; deg, degradation; Por, Porphyrin; CHLA, chlorophyll; GSH, Glutathione; GPL, Glycerophospholipid; ALA, alpha-Linoleic acid; ARA, arachidonic acid; SeC, Selenocompound; Alpha-AAA, alpha-aminoadipic acid; lysoPC, lysophosphocholine; PC, phosphocholine; SM, sphingomyelin.

While all fetuses included in this contrast were highly infected, the contrast highlighted metabolic changes associated with fetal compromise represented by the presence of meconium staining on skin of the MEC fetuses. In addition to the amino acids and sphingolipid metabolism, the citrate cycle and the arachidonic acid metabolism pathways influential in this contrast (Figures 4A,B). The metabolome of the MEC group was more homogeneous and distinct than the HVL-VIA group based on PLS-DA (2 components, *R*^2^ = 0.54, *Q*^2^ = 0.31) (*P* = 0.006; 13/2,000 permutations) (Figure 4C). Alpha-AAA, but not kynurenine, ranked the top 15 contributors to group separation (Figure 4D). While the top 15 contributors were comprised mainly of PCs and lysoPCs, many other metabolites including carnosine, glutamic acid, serotonin and succinate were significantly altered (FDR < 0.05) in the MEC group suggesting they are directly or indirectly associated with disease progression. Histamine was the only compound with higher concentration in the HVL-VIA group.

**Figure 5:**
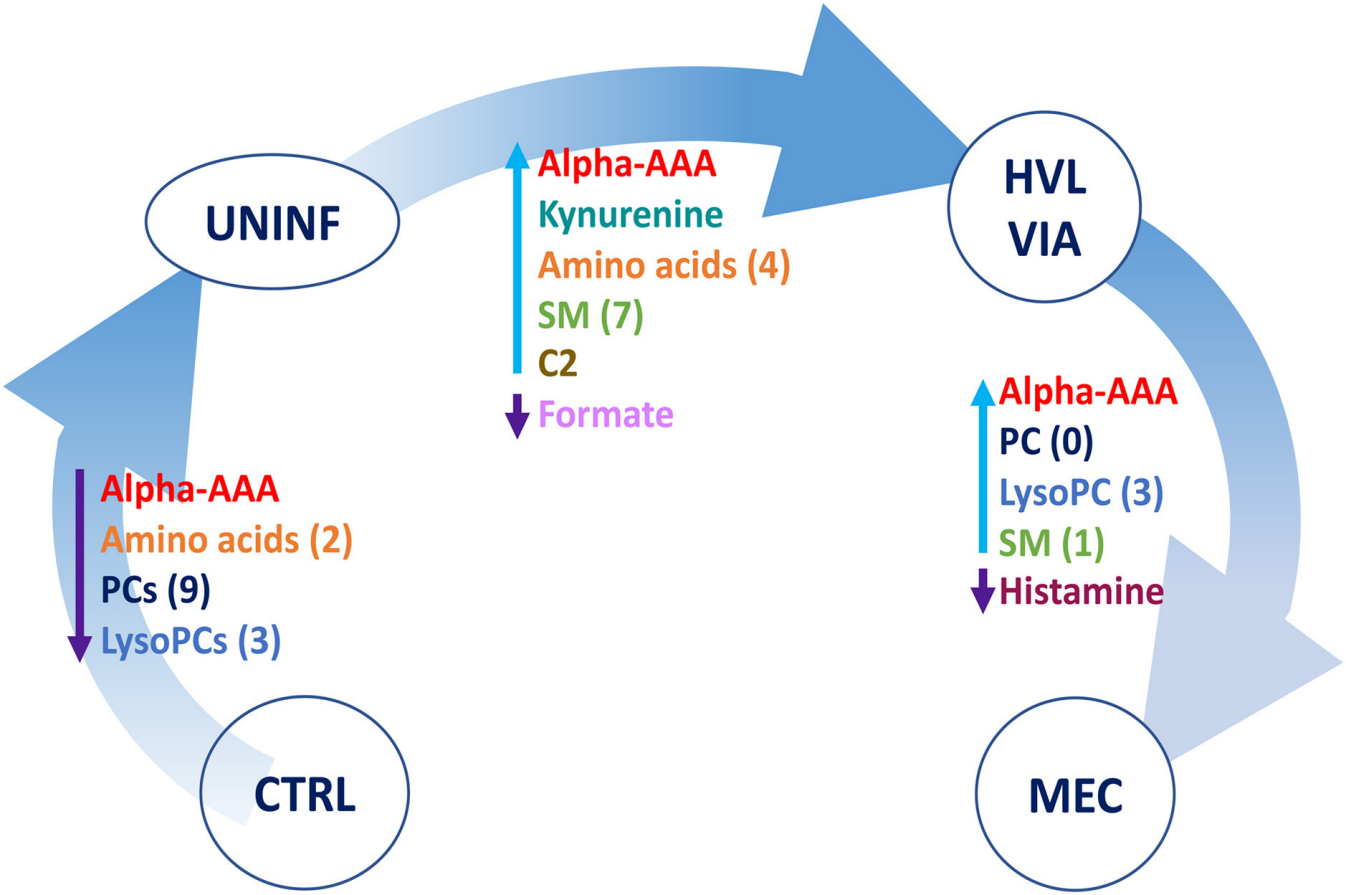
Visual summary of the most significant metabolite changes associated with disease progression. 1.CTRL: fetuses from non-inoculated control gilts; 2.UNINF: fetuses from PRRSV inoculated gilts that escaped infection, i.e., negative viral load; 3.HVL-VIA: viable PRRSV-infected fetuses with high viral load (over 3.5 log_10_; mean 6.7 ± 0.9 genome copies/mg) in fetal thymus; and 4.MEC: PRRSV-infected meconium-stained fetuses with high viral load (over 5 log_10_; mean 7.3 ± 0.9 genome copies/mg). Alpha-AAA, alpha-aminoadipic acid; ADMA, asymmetric dimethylarginine C2, acetylcarnitine; lysoPC, lysophosphocholine; PC, phosphocholine, SM, sphingomyelin.

A summary of these contrasts to highlight the most important metabolite changes associated with disease progression is shown in Figure 5.

### Metabolomic Profiles Associated With Altered Fetal Development and PRRSV Infection

**Figure 6:**
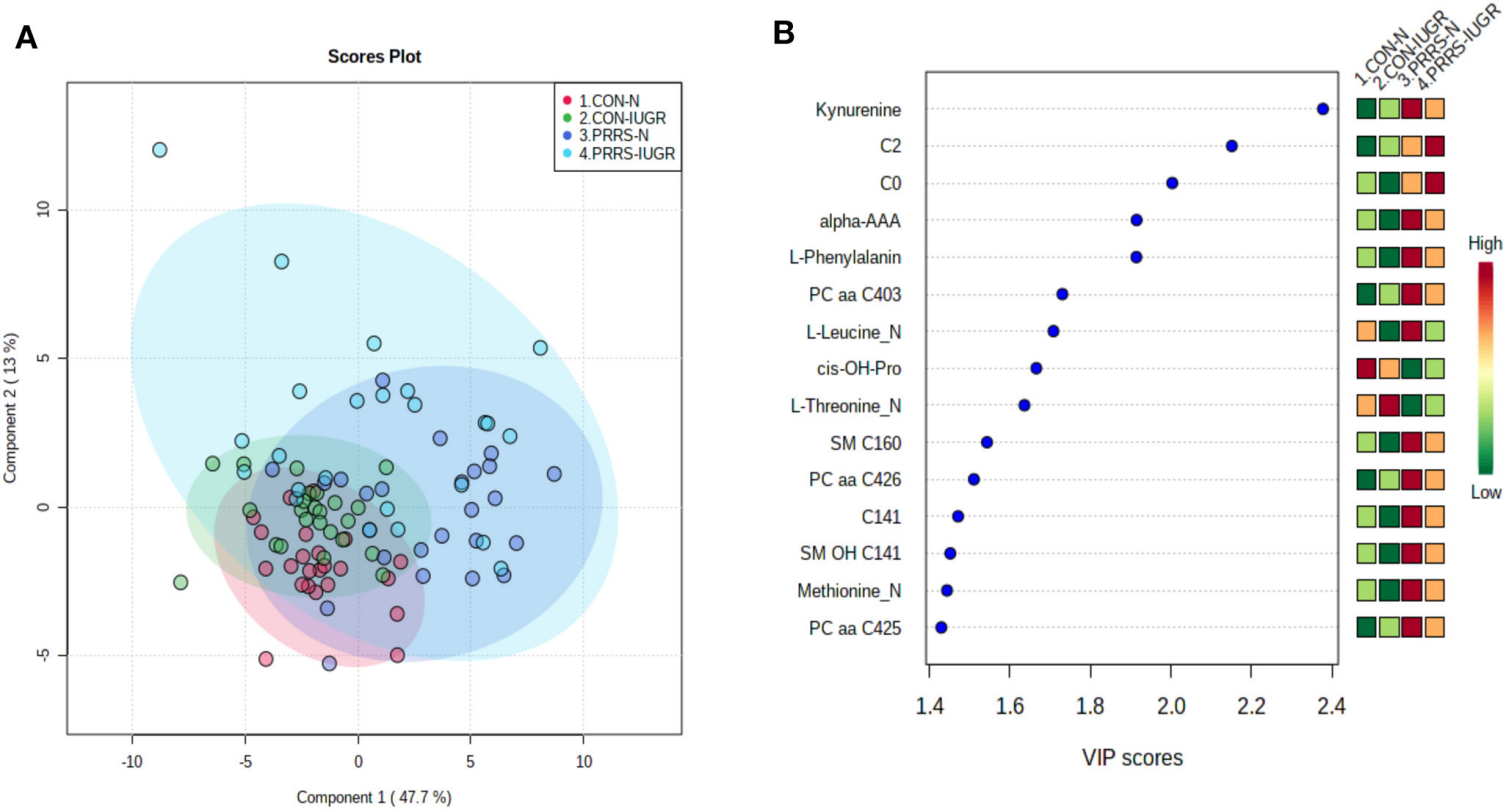
Metabolite profiles of four IUGR x PRRV-infected. **(A)** Two component PLS-DA score plot of all groups with individual fetuses represented by dots; **(B)** Variance Importance in Projection (VIP) score plot displaying the 15 most important metabolites differentiating the groups with colored side bar displaying the relative metabolite concentration in each group. 1.CON-N: non-IUGR (normal development) fetuses from non-inoculated control gilts; 2.CON-IUGR: IUGR fetuses from non-inoculated control gilts; 3.PRRS-N: non-IUGR fetuses from PRRSV-infected gilts; and 4.PRRS-IUGR: IUGR fetuses from PRRSV-infected gilts. Alpha-AAA, alpha-aminoadipic acid; C0, carnitine; C2, acetylcarnitine; *cis*-OH-Pro, cis-4-Hydroxy-L-proline; PC, phosphocholine; SM, sphingomyelin.

As previous research identified intrauterine growth rate as a predictor of infection, we next compared the global metabolome in a two by two experiment to investigate this relationship. In total, 141 common metabolites (DI-MS: 109, NMR: 32) were detected and 68 of these significantly differed (FDR < 0.01) among groups based on Kruskal-Wallis. Although there was substantial overlap among all four groups based on the PLS-DA analysis (3 components, *R*^2^ = 0.62, *Q*^2^ = 0.50), the metabolomes of non-infected (CON-N and CON-IUGR) fetuses were more homogeneous than the PRRSV-infected fetuses (Figure 6A) and significantly different from each other (*P* < 5e-04; 0/2,000 permutations). Across all four groups, the metabolites with highest VIP scores were mainly kynurenine, carnitines (C0 and C2), alpha-AAA, PCs and amino acids, being mostly higher in the PRRSV-infected fetuses (Figure 6B) and all presenting with VIP scores between 1.4 and 2.4. Specific contrasts were further investigated to elucidate the interaction between fetal development and infection status. Individual group contrasts are described in more detail below and Table 2 highlights all the VIP metabolites indicating their significance, VIP score, and fold change (FC) between groups for each contrast.

**Table 2:** Metabolites with significant differences between contrasting PRRSV-infected and IUGR phenotypic groups.

**Contrasts**	**CON-N vs. CON-IUGR**			**CON-N vs. PRRS-N**			**CON-IUGR vs. PRRS-IUGR**			**PRRS-N vs. PRRS-IUGR**		
**Metabolite**	**VIP score**	**FDR**	**FC**	**VIP score**	**FDR**	**FC**	**VIP score**	**FDR**	**FC**	**VIP score**	**FDR**	**FC**
Alpha-AAA	<1	ns	ns	1.76	<0.001	0.423	1.74	ns	0.430	1.16	0.042	ns
C0	<1	ns	ns	<1	0.002	ns	2.27	0.003	ns	<1	ns	ns
C141	<1	ns	ns	1.24	<0.001	ns	1.43	ns	ns	<1	0.045	ns
C2	<1	ns	ns	<1	0.016	ns	2.05	ns	ns	<1	ns	ns
*cis*-OH-Pro	<1	ns	ns	1.26	<0.001	ns	1.49	0.006	ns	<1	ns	ns
Glycine	1.17	ns	ns	<1	<0.001	0.470	1.36	ns	ns	<1	ns	ns
Histamine	<1	ns	ns	1.27	0.001	ns	<1	ns	ns	1.34	0.013	ns
Kynurenine	<1	ns	ns	1.08	<0.001	0.305	2.61	ns	0.248	<1	ns	ns
L-Alanine	1.30	0.005	ns	<1	0.006	ns	<1	ns	ns	<1	0.050	ns
L-Glutamine	2.04	<0.001	ns	<1	ns	ns	<1	ns	ns	<1	0.011	ns
L-Leucine	1.86	<0.001	ns	<1	ns	ns	1.51	ns	ns	1.31	ns	ns
L-Phenylalanine	<1	ns	ns	1.31	<0.001	ns	2.10	ns	ns	<1	ns	ns
L-Threonine	<1	ns	ns	<1	0.005	ns	1.75	0.036	ns	<1	ns	ns
LysoPC a c160	1.54	0.007	ns	<1	ns	ns	<1	ns	ns	1.38	0.010	ns
LysoPC a c161	1.31	ns	ns	<1	ns	ns	<1	ns	ns	1.14	0.026	ns
LysoPC a c170	1.56	0.004	ns	<1	ns	ns	<1	ns	ns	1.27	0.013	ns
LysoPC a c180	1.75	<0.001	ns	1.10	<0.001	ns	<1	ns	ns	1.70	0.008	ns
LysoPC a c181	1.62	0.009	ns	1.22	<0.001	ns	<1	ns	ns	1.58	0.010	ns
LysoPC a c182	1.76	0.007	ns	<1	0.026	ns	<1	ns	ns	1.47	0.010	ns
LysoPC a c203	1.45	ns	ns	1.36	<0.001	ns	1.40	ns	ns	1.37	0.019	ns
Methionine	<1	ns	ns	<1	0.004	ns	1.48	ns	ns	<1	ns	ns
PC aa C360	1.40	ns	ns	1.05	0.001	ns	<1	ns	ns	1.23	0.026	ns
PC aa C361	<1	ns	ns	1.36	<0.001	ns	<1	ns	ns	1.31	0.011	ns
PC aa C381	<1	ns	ns	1.36	<0.001	ns	<1	ns	ns	1.11	0.026	ns
PC aa C383	1.07	ns	ns	1.26	<0.001	ns	1.05	ns	ns	1.18	0.020	ns
PC aa C386	1.26	ns	ns	<1	0.003	ns	<1	ns	ns	1.43	0.011	ns
PC aa C403	<1	ns	ns	1.45	<0.001	0.494	1.14	ns	ns	<1	ns	ns
PC aa C404	<1	ns	ns	1.51	<0.001	0.490	<1	ns	ns	<1	ns	ns
PC aa C405	<1	ns	ns	1.48	<0.001	ns	<1	ns	ns	1.10	ns	ns
PC aa C406	<1	ns	ns	1.18	<0.001	ns	<1	ns	ns	1.35	0.014	ns
PC aa C424	<1	ns	ns	1.46	<0.001	0.494	<1	ns	ns	<1	ns	ns
PC aa C425	1.12	ns	ns	1.34	<0.001	ns	<1	ns	ns	<1	ns	ns
PC ae C423	1.43	0.021	ns	<1	0.004	ns	<1	ns	ns	1.29	0.019	ns
SM C160	<1	ns	ns	1.42	<0.001	ns	1.62	ns	ns	1.13	0.022	ns
SM C240	1.20	ns	ns	1.07	<0.001	ns	<1	ns	ns	1.20	0.014	ns
SM OH C141	<1	ns	ns	1.40	<0.001	ns	1.57	ns	ns	1.11	0.027	ns
SM OH C221	1.27	ns	ns	<1	0.002	ns	<1	ns	ns	1.29	0.011	ns
Tyrosine	<1	ns	ns	<1	ns	ns	2.15	0.036	ns	<1	0.026	ns
Valine	1.70	<0.001	ns	<1	ns	ns	<1	ns	ns	<1	ns	ns

*FC, fold change; FDR, false discovery rate; VIP, Variable Importance in Projection; ns, not significant*.

#### Control-Normal vs. Control-IUGR

**Figure 7:**
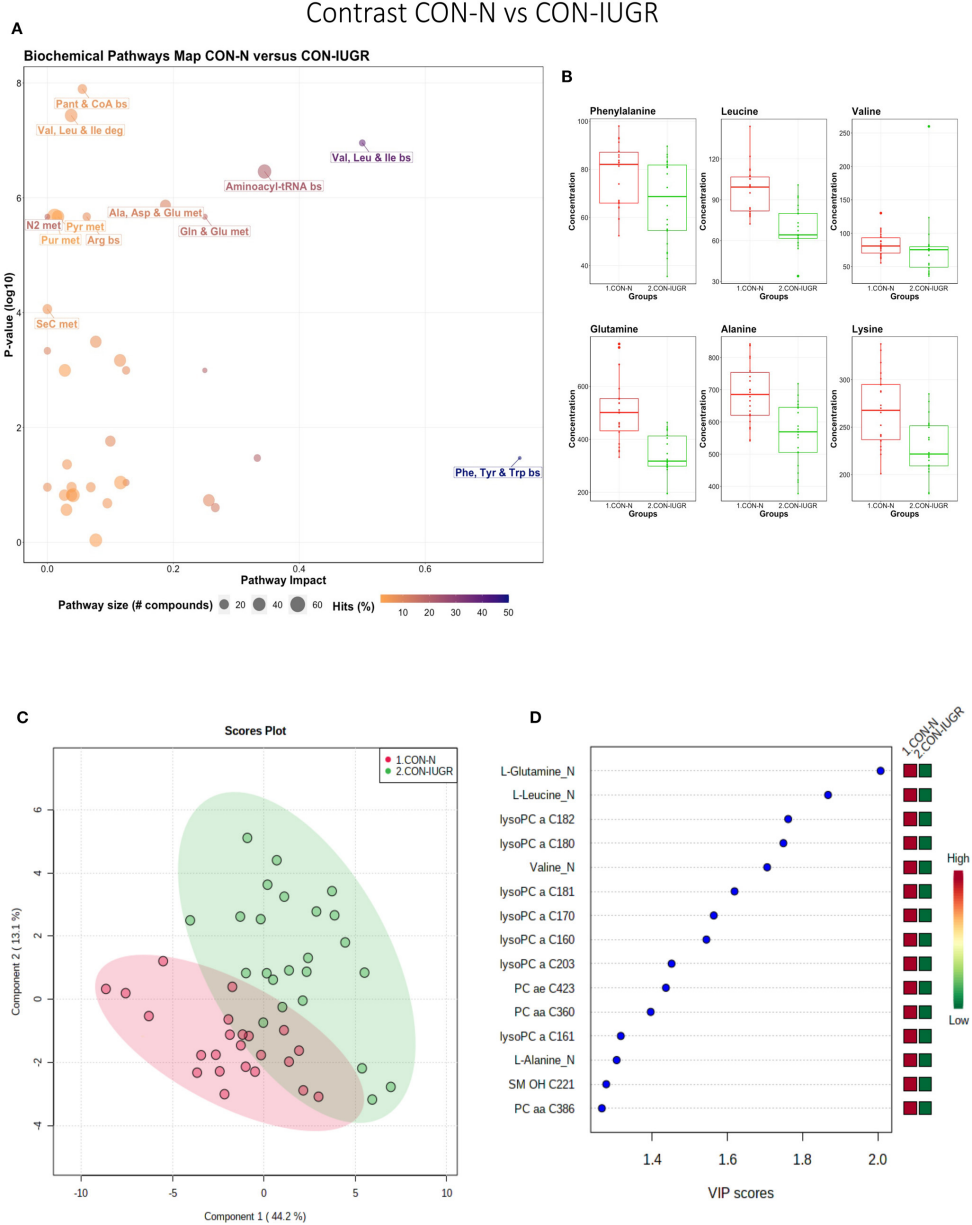
Metabolite profile of control-normal development fetuses (CON-N) and control-IUGR fetuses (CON-IUGR). **(A)** Biochemical pathway plot displaying the most significant (Y-axis; *P*-value < 0.05) and impactful (X-axis) pathways distinguishing the groups. Size of dot represents the size of the pathway (number of compounds). Dark colors (from light orange to dark purple) represent higher hits (percentage of the pathway detected in our study). **(B)** Boxplots of the concentration of significant (*P*-value < 0.05) compounds distinguishing the groups. **(C)** Two component PLS-DA score plots with individual fetuses represented by dots. **(D)** Variance Importance in Projection (VIP) score plots displaying the 15 most important metabolites differentiating groups with colored side bar displaying the relative metabolite concentration in each group. 1.CON-N: non-IUGR (normal development) fetuses from non-inoculated control gilts; 2.CON-IUGR: IUGR fetuses from non-inoculated control gilts. Met, metabolism; bs, biosynthesis; deg, degradation; Por, Porphyrin; CHLA, chlorophyll; GSH, Glutathione; GPL, Glycerophospholipid; ALA, alpha-Linoleic acid; ARA, arachidonic acid; SeC, Selenocompound; LysoPC, lysophosphocholine; PC, phosphocholine; SM, sphingomyelin.

This contrast aimed to display metabolomic differences between normal and IUGR fetuses (disparaging fetal development) prior to infection. The most influential pathways in this contrast related to amino acid metabolism or biosynthesis (Figures 7A,B). These groups were significantly different (*P* < 5e-04; 0/2,000 permutations) based on the PLS-DA analysis (three components, *R*^2^ = 0.79, *Q*^2^ = 0.55) (Figure 7C) and most metabolites were at greater concentration in the non-IUGR compared to IUGR fetuses, likely commensurate with greater growth rates. Metabolites contributing to group differences were amino acids (glutamine, leucine, valine, alanine), lysoPCs (7), PCs (3) and SM (1); all were increased in the non-IUGR fetuses (Figure 7D).

#### Control-Normal vs. PRRS-Normal

**Figure 8:**
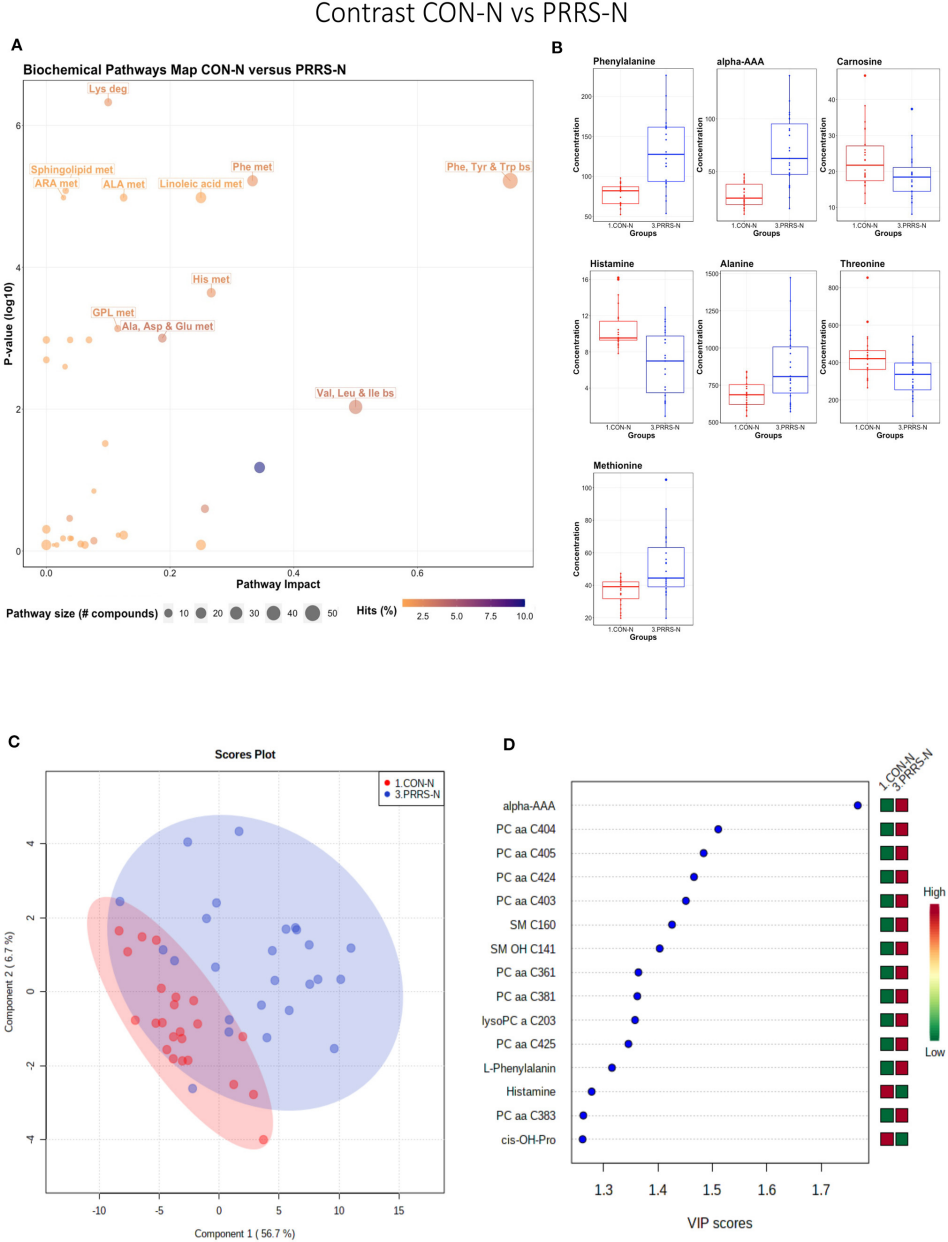
Metabolite profile of control-normal development fetuses (CON-N) and PRRS-infected normal development fetuses (PRRS-N). **(A)** Biochemical pathway plot displaying the most significant (Y-axis; *P*-value < 0.05) and impactful (X-axis) pathways distinguishing the groups. Size of dot represents the size of the pathway (number of compounds). Dark colors (from light orange to dark purple) represent higher hits (percentage of the pathway detected in our study). **(B)** Boxplots of the concentration of significant (*P*-value < 0.05) compounds distinguishing the groups. **(C)** Two component PLS-DA score plots with individual fetuses represented by dots. **(D)** Variance Importance in Projection (VIP) score plots displaying the 15 most important metabolites differentiating groups with colored side bar displaying the relative metabolite concentration in each group. 1.CON-N: non-IUGR (normal development) fetuses from non-inoculated control gilts; 3.PRRS-N: non-IUGR fetuses from PRRSV-infected gilts. Met, metabolism; bs, biosynthesis; deg, degradation; Por, Porphyrin; CHLA, chlorophyll; GSH, Glutathione; GPL, Glycerophospholipid; ALA, alpha-Linoleic acid; ARA, arachidonic acid; SeC, Selenocompound; Alpha-AAA, alpha-aminoadipic acid; *cis*-OH-Pro, *cis*-4-Hydroxy-L-proline; lysoPC, lysophosphocholine; PC, phosphocholine; SM, sphingomyelin.

This contrast highlighted differences between control and infected fetuses exhibiting normal fetal development. The most impactful pathways in this contrast related to amino acids sphingolipids and arachidonic acid metabolism (Figures 8A,B). Not surprisingly, the groups were significantly different (*P* < 5e-04; 0/2,000 permutations) based on the PLS-DA analysis (Figure 8C; 2 components, *R*^2^ = 0.60, *Q*^2^ = 0.40) and similar to the infected vs. non-infected (CTRL × UNINF, UNINF × HVL-VIA) contrasts of the disease progression experiment described above (Figures 2, 3), but with important differences. Firstly, kynurenine was not a prominent metabolite distinguishing group (Figure 8D) where it had the second highest VIP score in UNINF × HVL-VIA contrast. Secondly, phenylalanine was the only amino acid with a high VIP score in this contrast whereas there were five amino acids with high VIP scores in the CTRL × UNINF and UNINF × HVL-VIA comparisons. Alpha-AAA scored prominently in all, highlighting it as a major biomarker of PRRSV infection of the fetus, being consistently elevated in fetal sera following infection.

#### Control-IUGR vs. PRRS-IUGR

**Figure 9:**
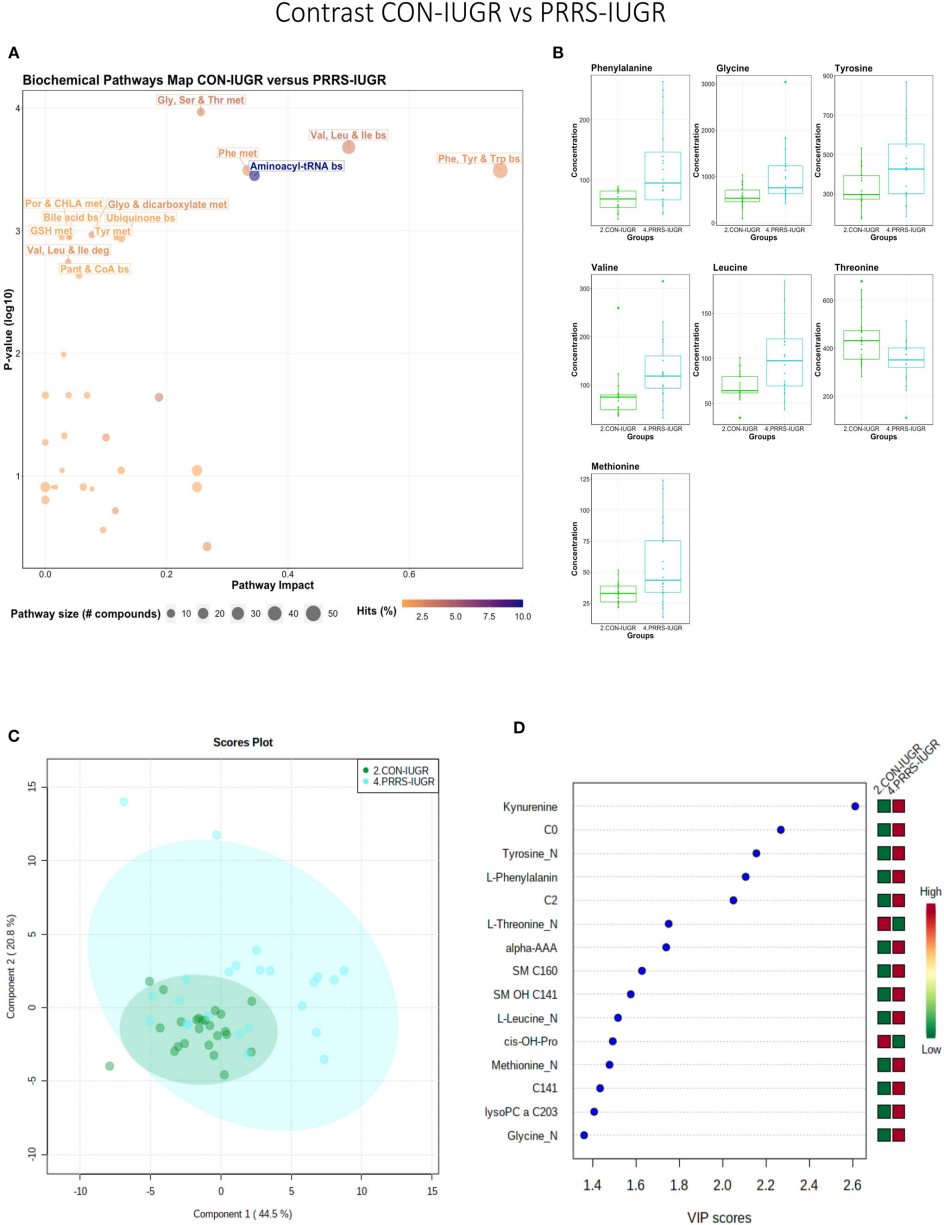
Metabolite profile of control-IUGR (CON-IUGR) and PRRS-infected IUGR fetuses (PRRS-IUGR). **(A)** Biochemical pathway plot displaying the most significant (Y-axis; *P*-value < 0.05) and impactful (X-axis) pathways distinguishing the groups. Size of dot represents the size of the pathway (number of compounds). Dark colors (from light orange to dark purple) represent higher hits (percentage of the pathway detected in our study). **(B)** Boxplots of the concentration of significant (*P*-value < 0.05) compounds distinguishing the groups. **(C)** Two component PLS-DA score plots with individual fetuses represented by dots. **(D)** Variance Importance in Projection (VIP) score plots displaying the 15 most important metabolites differentiating groups with colored side bar displaying the relative metabolite concentration in each group. 2.CON-IUGR: IUGR fetuses from non-inoculated control gilts; 4.PRRS-IUGR: IUGR fetuses from PRRSV-infected gilts. Met, metabolism; bs, biosynthesis; deg, degradation; Por, Porphyrin; CHLA, chlorophyll; GSH, Glutathione; GPL, Glycerophospholipid; ALA, alpha-Linoleic acid; ARA, arachidonic acid; SeC, Selenocompound; C0, carnitine; C2, acetylcarnitine; C141, tetradecenoyl carnitine; *cis*-OH-Pro, *cis*-4-Hydroxy-L-proline; lysoPC, lysophosphocholine; SM, sphingomyelin.

This contrast aimed to display the metabolic differences in response to infection in the fetal IUGR population. A number of amino acid related pathways were altered between these groups (Figure 9A) with amino acid levels typically greater in the PRRSV-infected group (Figure 9B). Group differences were significant (*P* = 0.001; 3/2,000 permutations) based on the PLS-DA analysis (three components, *R*^2^ = 0.55, *Q*^2^ = 0.26), possibly related to the extremely diverse global metabolome of the PRRS-IUGR group (Figure 9C) or that the PRRS response in IUGR fetuses was fundamentally different than the normally developed cohorts. Six of the top 15 VIP score metabolites distinguishing these groups were amino acids. Others included carnitines C0, C2, C14:1) and some lysoPCs and SMs (Figure 9D). Kynurenine had the highest VIP score, while alpha-AAA ranked 7th, scoring under 2.

#### PRRS-Normal vs. PRRS-IUGR

**Figure 10:**
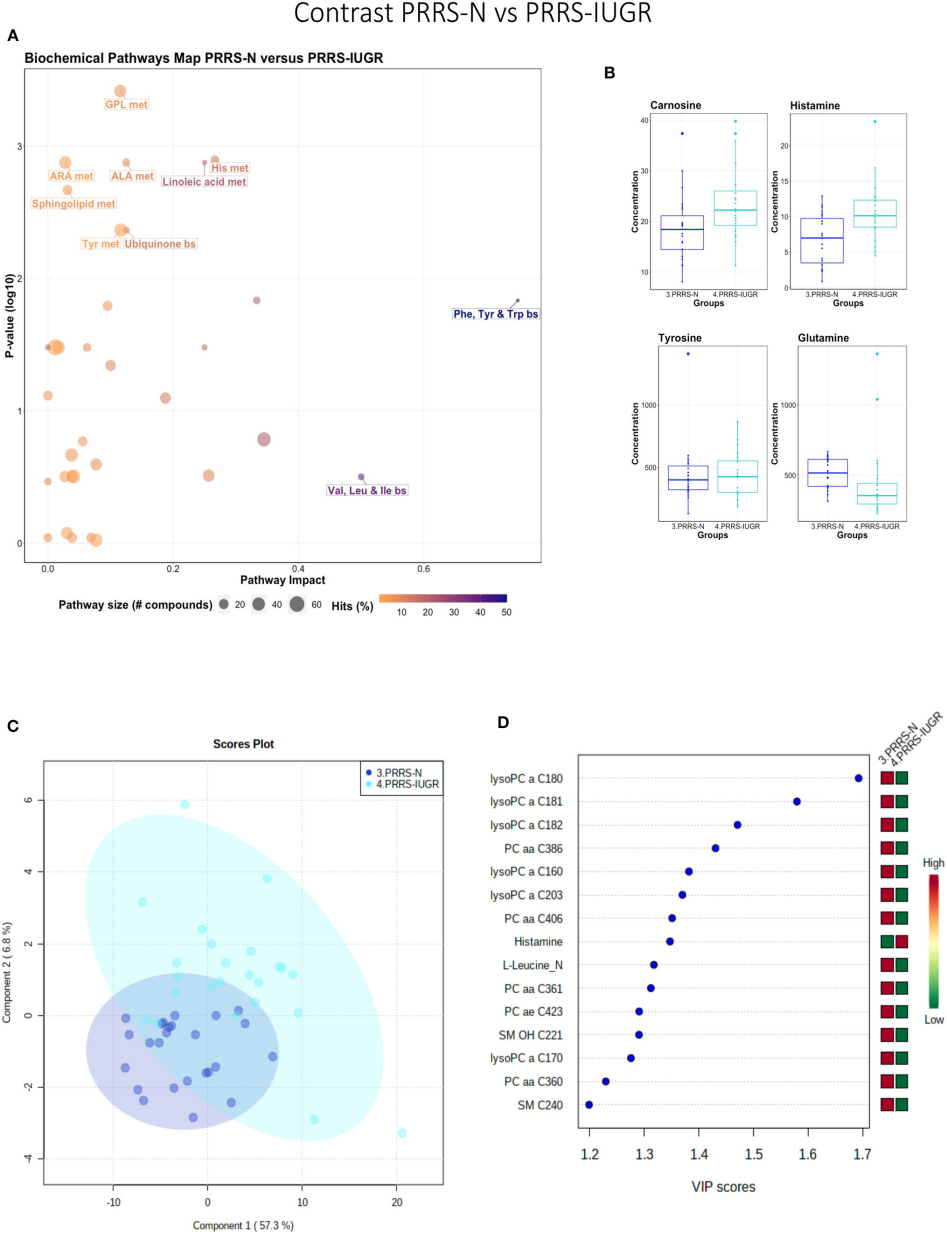
Metabolite profile of PRRS-infected normal development fetuses (PRRS-N) and PRRS-infected IUGR fetuses (PRRS-IUGR). **(A)** Biochemical pathway plot displaying the most significant (Y-axis; *P*-value < 0.05) and impactful (X-axis) pathways distinguishing the groups. Size of dot represents the size of the pathway (number of compounds). Dark colors (from light orange to dark purple) represent higher hits (percentage of the pathway detected in our study). **(B)** Boxplots of the concentration of significant (*P*-value < 0.05) compounds distinguishing the groups. **(C)** Two component PLS-DA score plots with individual fetuses represented by dots. **(D)** Variance Importance in Projection (VIP) score plots displaying the 15 most important metabolites differentiating groups with colored side bar displaying the relative metabolite concentration in each group. 3.PRRS-N: non-IUGR fetuses from PRRSV-infected gilts; 4.PRRS-IUGR: IUGR fetuses from PRRSV-infected gilts. Met, metabolism; bs, biosynthesis; deg, degradation; Por, Porphyrin; CHLA, chlorophyll; GSH, Glutathione; GPL, Glycerophospholipid; ALA, alpha-Linoleic acid; ARA, arachidonic acid; SeC, Selenocompound; LysoPC, lysophosphocholine; PC, phosphocholine; SM, sphingomyelin.

This contrast highlighted differences between normal and IUGR fetuses after maternal PRRSV infection. The diverging pathways between these two groups were mainly related to amino acid metabolism, as well as sphingolipids and arachidonic acid metabolism (Figure 10A), however, only four amino acids differed significantly between group (Figure 10B), lower than other the previous contrasts. Whereas, the IUGR vs. non-IUGR contrast in control fetuses found three amino acids among the prominent metabolites distinguishing group, only one amino acid scored in the top 15 metabolites distinguishing non-IUGR and IUGR fetuses following maternal PRRSV infection (Figures 10C,D). Group separation and differences (*P* = 0.001; 2/2,000 permutations) by PLS-DA analysis (four components, *R*^2^ = 0.73, *Q*^2^ = 0.54) were mainly related to differences in lysoPCs, PCs, and SM concentration, all decreased in the PRRS-IUGR group, with the exception of histamine and *cis*-4-Hydroxy-L-proline (*cis*-OH-Pro).

### Exploring PRRS IUGR Group Diversity

**Figure 11:**
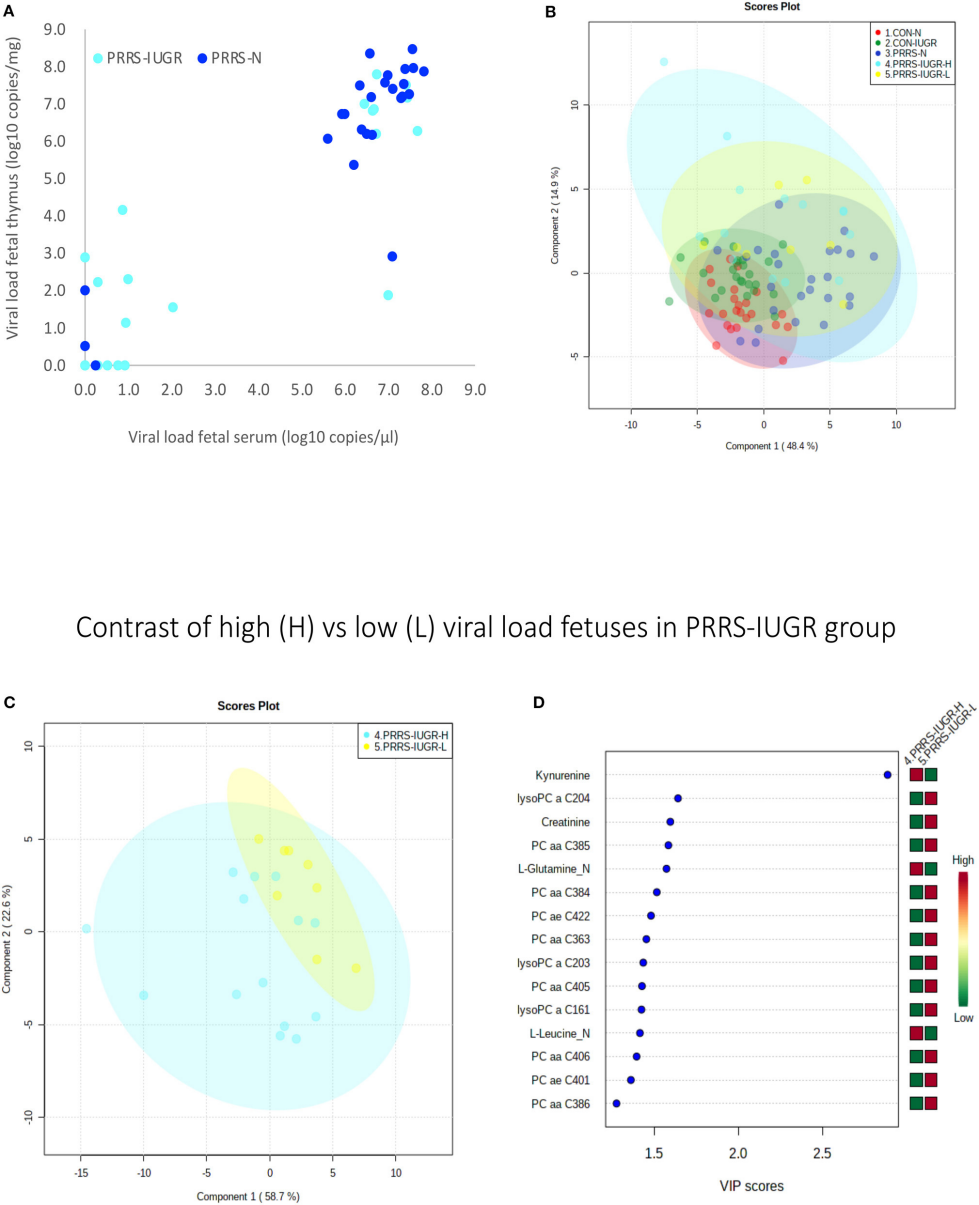
Analysis of the PRRSV-IUGR group as two subgroups based on PRRS viral concentration. **(A)** PRRSV viral load in thymus and serum of PRRSV infected IUGR and non-IUGR/normal (N) fetuses. **(B)** Two component score plot PLS-DA plot of all PRRS x IUGR groups with PRRS-IUGR subdivided into high (H) and low (L) viral load subgroups. **(C)** Two component PLS-DA score plots of the PRRS-IUGR high (H) and low (L) sub-groups only. **(D)** Variance Importance in Projection (VIP) score plots displaying the 15 most important metabolites differentiating high and low viral load groups with colored side bar displaying the relative metabolite concentration in each group. 1.CON-N: non-IUGR (normal development) fetuses from non-inoculated control gilts; 2.CON-IUGR: IUGR fetuses from non-inoculated control gilts; 3.PRRS-N: non-IUGR fetuses from PRRSV-infected gilts; 4.PRRS-IUGR-H: high viral load subgroup; and 5.PRRS-IUGR-L: low/negative viral load subgroup. lysoPC, lysophosphocholine; PC, phosphocholine.

The wide heterogeneity of the PRRS-IUGR group prompted further investigation to determine if it was associated with viral infection. While all PRRS-N fetuses were PRRSV-infected and the majority (22/25) had high viral concentration in fetal serum and thymus, the PRRS-IUGR group was comprised of fetuses with high (10/24), low (9/24) or non-detectable (5/24) viral load in sera or thymus (Figure 11A), largely because IUGR fetuses are protected from PRRSV infection (Ladinig et al., 2014a) and were sparse. After splitting the PRRS-IUGR into high (PRRS-IUGR-H) and low/negative (PRRS-IUGR-L) viral load subgroups, 78 metabolites significantly differed (FDR < 0.01) amongst the five groups based on Kruskal-Wallis testing and group differences were significant based on PLS-DA analysis (three components, *R*^2^ = 0.60, *Q*^2^ = 0.40) (*P* < 5e-04; 0/2,000 permutations)(Figure 11B). However, no significant (*P* = 0.351; 703/2,000 permutations) differences between the high and low viral load subgroups were evident on the two-group PLS-DA contrast (3 components, *R*^2^ = 0.63, *Q*^2^ = 0.16) (Figure 11C). Kynurenine was the most distinguishing metabolite being higher in PRRSV-IUGR-H fetuses (Figure 11D) corresponding to higher levels of viral infection. There was a variety of other metabolites with high VIP scores including leucine and glutamine (increased in PRRS-IUGR-H subgroup), while creatinine and several lysoPC/PCs were all increased in PRRS-IUGR-L subgroup.

**Figure 12:**
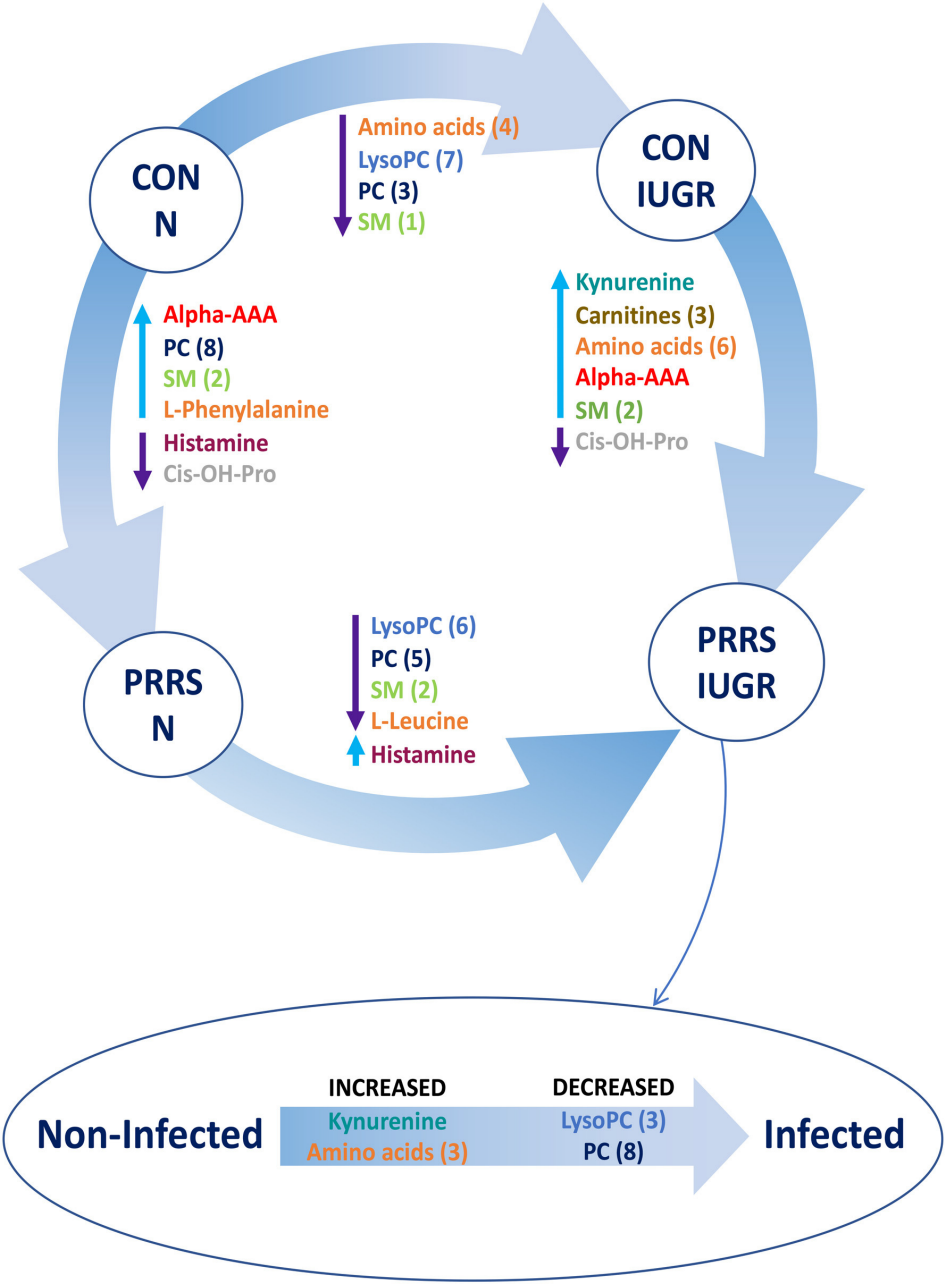
Visual summary of the most significant metabolite changes associated with PRRSV-infection of fetuses with normal development or intrauterine growth retardation (IUGR). 1.CON-N: non-IUGR (normal development) fetuses from non-inoculated control gilts; 2.CON-IUGR: IUGR fetuses from non-inoculated control gilts; 3.PRRS-N: non-IUGR fetuses from PRRSV-infected gilts; and 4.PRRS-IUGR: IUGR fetuses from PRRSV-infected gilts. Alpha-AAA, alpha-aminoadipic acid; *cis*-OH-Pro, *cis*-4-Hydroxy-L-proline; lysoPC, lysophosphocholine; PC, phosphocholine; SM, sphingomyelin.

A summary of the most important metabolite alterations related to the interaction of PRRSV-infection and IUGR is shown in Figure 12.

## Discussion

This global metabolomics profiling utilized archived fetal serum to explore the progression of disease and the impact of fetal development following PRRSV infection. To our knowledge, this is the first research describing alterations in the global metabolome of fetal pigs during maternal PRRSV infection and one of the first studies to investigate fetuses experiencing IUGR which are naturally resilient to prenatal PRRSV infection. As such, it provides novel insights into PRRS virus infection as well as for infective diseases in pigs and other livestock species.

An important initial finding with respect to PRRS disease progression is that most of the VIP metabolites were at high levels in control fetuses (CTRL), lower in UNINF fetuses, and increased again in the PRRSV-infected groups (HVL-VIA and MEC). With respect to the interaction of PRRSV and fetal development, higher metabolite levels were observed in non-IUGR vs. IUGR fetuses as well as in PRRSV-infected vs. non-infected groups. These results suggest fetal responses are influenced by both factors.

Following maternal PRRSV-challenge, virus quickly infects the uterine tissues, then transmits across maternal uterine and fetal trophoblast epithelial layers to placental tissue and fetal circulation (Malgarin et al., 2019). The rate of transplacental transmission varies and some fetuses escape infection while others succumb to infection resulting in varied fetal outcomes along a susceptibly-resilience spectrum (Harding et al., 2017). UNINF fetuses are most resilient and dead fetuses most susceptible. In the present research, however, dead fetuses were not included because it was not possible to collect high quality blood samples. Thus, our proxy for susceptible fetuses were those affected by meconium staining of skin, a clinical sign associated with PRRS disease progression (Ladinig et al., 2014d), and imminent death in the most severe cases.

In spite of being more resilient to PRRSV-infection, UNINF fetuses responded to maternal infection in agreement with past research showing differentially expressed genes related to innate and inflammatory responses in fetal thymus following infection of the endometrium (Wilkinson et al., 2016). One of the most important metabolites distinguishing UNINF from CTRL fetuses was alpha-AAA. Alpha-AAA was also a predominant metabolite in other PRRSV-infected groups (HVL-VIA, PRRS-N, PRRS-IUGR) compared to non-infected (CTRL, CON-N, CON-IUGR). It is a key compound in the lysine biosynthesis and degradation pathways. A number of amino acid biosynthesis and degradation metabolism pathways were also significantly altered between the PRRSV-infected and non-infected groups indicating a disruption in amino acids' pathways or the energy cycle (tricarboxylic acid cycle; TCA).

Although it is not yet completely understood how PRRSV crosses the maternal-fetal interface (MFI) to infect fetuses (Karniychuk and Nauwynck, 2013), IUGR fetuses have lower viral loads than fetuses with normal intrauterine growth (non-IUGR) (Ladinig et al., 2014a; Malgarin et al., 2019). Intrauterine growth retardation in swine may result from many genetic, epigenetic, maternal and environmental factors as previously reviewed (Wu et al., 2007). Impaired placental growth is one of the most important factors associated with IUGR, which has a negative impact on placental size and blood flow, resulting in a lower nutrient transport to the fetus compared to its regular sized siblings. This likely explains the lower levels of metabolites in the IUGR groups in this study. It is also possible that virus enters the fetal compartment from the dam by transiting through the maternal and fetal epithelial layers. In fact, PRRSV is capable of entering and exiting trophoblastic epithelium *in vitro* by vesicle-mediated intercellular communication (Suleman et al., 2019), supporting this hypothesis. If fetal infection does rely on mechanisms of intercellular communication inherent to epithelial cells, the placental inefficiency underlying fetal growth retardation may be a protective factor against PRRSV transplacental infection. Consistent with placental inefficiency, many lysoPCs and PCs were decreased in UNINF, CON-IUGR and PRRS-IUGR fetuses. Biosynthesis of these compounds can be regulated by the availability of certain amino acids (Havener and Toback, 1980). The lower amino acid levels (possibly some essential) in these groups compared to CON and non-IUGR groups might result in lower levels of the PCs and lyso PCs as well. Besides their natural biochemical pathway (glycerophospholipid metabolism) (Holmsen et al., 1992), lysoPCs and PCs can be used to synthesize arachidonic acid in response to tumor necrosis factor alpha (TNF-alpha). Arachidonic acid metabolism, which is significantly different between groups in most contrasts, is the precursor of eicosanoids, like prostaglandin E, among many others (Hanna and Hafez, 2018) that can work as pro-inflammatory molecules and the lack of those compounds might serve as a protective factor for the UNINF and IUGR fetuses or may reflect the lack of nutrition.

Several contrasts in the present study (UNINF vs. HVL-VIA, CTRL-N vs. PRRS-N, CON-IUGR vs. PRRS-IUGR) demonstrate the fetus's first reaction to PRRSV-infection. While alpha-AAA was increased in fetuses following PRRSV-infection, kynurenine was the predominant metabolite increased in HVL-VIA fetuses. Kynurenine is a biproduct of tryptophan metabolism regulated by the enzyme Indoleamine 2,3-Dioxygenase (IDO1) (Takikawa et al., 1986). IDO1 can be induced by increased levels of TNF-alpha, IFN-gamma and IFN-beta (King and Thomas, 2007; Wilkinson et al., 2016), cytokines normally elevated in response to PRRSV infection (Rowland, 2010; Ladinig et al., 2014c; Pasternak et al., 2020). Thus, the high levels of kynurenine would be expected following fetal infection and may be a potential biomarker for differentiating resilient from susceptible fetuses following PRRSV infection. Although alpha-AAA does not appear in the main metabolic pathways related to response to infection, its high concentration in PRRSV-infected groups implies it may be a potential biomarker to differentiate uninfected/control from infected fetuses.

The SM group of compounds was increased in the PRRSV-infected fetuses, and sphingolipid metabolism pathway significantly impacted in a number of groups contrasts. These compounds are part of lipid rafts on cell membranes, which are involved in PRRSV cell entry, replication and release in the host (Yang et al., 2015). Thus, the elevated level of SMs might be a response to infection, causing a high recruitment of these compounds. Alternatively, as these are important to the viral replication cycle in the host and can change the availability and function of the receptor CD163 (Yang et al., 2015), animals with greater concentration of SMs on their cell surfaces may be more susceptible to infection.

The contrast HVL-VIA vs. MEC reflects progression of disease to the point of compromise prior to fetal death. Most of the significantly different metabolites increased in MEC fetuses were PCs and SMs. SMs are compounds that crosslink a PC to ceramide (Gault et al., 2010). During inflammation, increased levels of TNF-alpha activate the sphingomyelinase enzyme (SMase) which induces the breakdown of the SMs releasing ceramides (Hannun and Obeid, 2008) and PCs, explaining the higher levels of PCs observed in PRRSV-infected fetuses. Ceramides can play a key role in activating apoptosis and recently have also been linked to necroptosis (programmed cell necrosis) (Hannun and Obeid, 2008; Chan et al., 2015). Thus, activation of this pathway during a sustained inflammatory response would eventually lead to apoptosis of fetal cells, compromising fetal tissues.

Histamine was the only metabolite that was decreased in MEC compared to HVL-VIA fetuses. Histamine is a well-known compound involved in allergic responses which can lead to anaphylactic reactions and is released by mast cells in most tissues when in contact with an antigen (Thurmond et al., 2008). It has been shown that mast cells can release histamine during an early primary response to viral infection (Graham et al., 2013). While histamine was significantly decreased in MEC compared to HVL-VIA fetuses, it was also numerically increased in HVL-VIA vs. UNINF fetuses consistent with a response to PRRSV infection and highlighting its potential as a biomarker of fetal PRRSV infection.

Other contrasts and comparisons could be investigated using different combinations of the groups, however, we opted to focus on disease progression (uninfected to infected to high viral load to meconium staining in a stepwise manner) as per our objective for this study. Moreover, several other metabolites were significantly altered in specific contrasts, however, not all had a plausible biological link to PRRSV infection or fetal development. In spite of an extensive literature search, the mechanisms underlying some of the metabolite alterations observed in the phenotypic contrasts described herein were not readily apparent. Such examples include acetic acid, ethanol, glutamine, creatinine, and carnitine in the contrast between PRRS-IUGR H and -L groups. Thus, we have limited our discussion to the metabolic alterations we believe provide a meaningful addition to the current knowledge of PRRS pathogenesis. It's also important to acknowledge the limitations of the AbsoluteIDQ® p180 Kit used in this study, which is limited to detect only the groups of compounds included in the kit (comprised of 180 analytes in total). However, the kit was designed to be a reliable and robust tool to detect and quantify a broad spectrum of metabolites. Although the biochemical pathway maps provide a general vision of the differences between groups, it is important to acknowledge they are limited to the kit's targeted detection system.

## Conclusions

For the first time, differences in the fetal metabolome of PRRSV-infected and non-infected fetuses were investigated. Clear differences were observed between contrasting resilience phenotypes, helping to elucidate mechanisms involved in fetal compromise and death. The lower levels of amino acids in UNINF fetuses possibly suggest a lower placental efficiency of these animals, which might protect them from PRRSV infection. Increased alpha-AAA and kynurenine levels in HVL-VIA fetuses are potential markers of susceptibility to PRRSV infection. The increased concentration of PCs in MEC fetuses may be a result of the degradation of the SMs and production of ceramides, and may be a marker of the compromised animals after viral infection. Fetuses experiencing normal *in utero* growth appear to have an increased level of lysoPCs, PCs and amino acids compared to IUGR fetuses, which might indirectly reflect greater intercellular communication across the maternal-fetal interface leading to higher risk of viral infection, while the almost complete absence of lysoPCs and PCs, even during infection, in the IUGR animals may indicate a different and perhaps muted response to infection associated with fetal growth retardation. These results bring novel insights into some possible mechanisms occurring in fetuses at various stages following maternal viral infection as well as elucidating new potential markers of fetal resilience to PRRSV infection.

## Data Availability Statement

The data has been uploaded to a public repository: www.ebi.ac.uk/metabolights/MTBLS2249.

## Ethics Statement

The animal study was reviewed and approved by University of Saskatchewan's Animal Research Ethics Board and adhered to the Canadian Council on Animal Care guidelines for humane animal use (permit #20110102).

## Author Contributions

CM: statistical analysis, data analysis, pathway investigation, and data interpretation. JH: experiment design and sample collection, statistical analysis, and data interpretation. DM: contributed to interpretation of results and approved the manuscript. All authors contributed to the article and approved the submitted version.

## Conflict of Interest

The authors declare that the research was conducted in the absence of any commercial or financial relationships that could be construed as a potential conflict of interest. The reviewer AL declared a past co-authorship with one of the authors JH to the handling editor.
